# NovelHTI: An Interpretable Pathway-Enhanced Framework for De Novo Target Prediction of Medicinal Herbs via Cross-Scale Heterogeneous Information Fusion

**DOI:** 10.3390/ph19030413

**Published:** 2026-03-03

**Authors:** Yuyam Cheung

**Affiliations:** Shanghai Key Laboratory of PMMP, School of Mathematical Sciences, East China Normal University, Dongchuan Rd. 500, Shanghai 200241, China; 52275500001@stu.ecnu.edu.cn

**Keywords:** systems pharmacology, target de-risking, herbal polypharmacology, phenotype-to-target, Mechanism of Action (MoA), Traditional Chinese Medicine modernization

## Abstract

**Background:** The modernization of Traditional Chinese Medicine (TCM) is hindered by a “structure-blind” bottleneck: establishing molecular mechanisms for complex formulations with uncharacterized chemical constituents. Conventional computational screening fails in these scenarios due to a heavy reliance on pre-determined structures. We developed NovelHTI, an inductive graph-based framework designed to reverse-engineer protein targets directly from standardized clinical symptom profiles. **Methods:** NovelHTI implements a “Phenotype-to-Target” paradigm by integrating heterogeneous graph neural networks with systemic pathway constraints. Unlike traditional transductive models, NovelHTI leverages multi-view feature fusion of symptom semantics and biological pathways to enable de novo prediction for unseen herbs. The framework was evaluated across 698 herbs and 7854 targets, benchmarking against advanced GNNs (HAN) and non-graph classifiers (XGBoost) under strict cold-start and knowledge erosion simulations. **Results:** NovelHTI maintains high precision (>84%) and balanced performance (F1-score >77%), outperforming baselines by over 33% (ROC-AUC) in realistic imbalanced screening, where traditional models typically fail (AUC ≈ 0.51). Robustness analysis confirmed stable performance (>0.83 AUC) despite 30% structural data incompleteness. Notably, retrospective validation successfully “rediscovered” emerging mechanisms (e.g., the Artemisinin-GPX4 ferroptosis axis) elucidated in 2021–2024 literature, which were entirely latent in the training data. **Conclusions:** NovelHTI provides a robust computational prioritization filter that effectively bridges macroscopic phenotypes and microscopic pharmacology. By enabling mechanism-driven target de-risking, this framework optimizes resource allocation for downstream experimental validation and accelerates TCM-based drug discovery.

## 1. Introduction

Natural products, particularly those documented in Traditional Chinese Medicine (TCM), represent a premier reservoir of chemical diversity for modern drug discovery [1,2,3]. With over 8000 recorded medicinal herbs, these resources function as sophisticated multi-component systems that modulate physiological networks through **polypharmacological mechanisms**, which is the simultaneous modulation of multiple targets and biological pathways [1]. Unlike the one-drug-one-target paradigm often seen in Western synthetic agents, the therapeutic efficacy of herbal medicines typically arises from the **synergistic interactions** of their constituents acting upon multiple targets [4]. However, this inherent complexity poses a substantial hurdle for modern pharmacognosy: the lack of a standardized framework to decode the holistic pharmacological profile of herbal mixtures severely impedes the systematic identification of their molecular targets.

Current strategies for target deconvolution largely depend on experimental approaches such as component isolation followed by high-throughput screening. While effective, this reductionist pipeline is labor-intensive, costly, and often fails to capture the “**herb-as-a-whole**” therapeutic effects derived from component synergy [5,6,7]. Recent studies in *Pharmaceuticals* and related journals have further highlighted that computational tools integrating multi-scale biological knowledge are critical for bridging traditional herbal medicine with modern drug discovery protocols [8,9]. However, large-scale pharmacological characterization of unstudied herbs remains impractical due to the lack of such specialized frameworks [2,10]. There is, therefore, a critical unmet need for computational frameworks capable of addressing molecular-level resolution constraints by generating **holistic herb representations**. To be practically valuable in a pharmaceutical context, such models must facilitate ***de novo** ***target prediction** for “novel herbs”, those without prior interaction records, thereby accelerating the mining of vast, untapped natural resources [11,12,13,14].

Beyond the specific domain of ethnopharmacology, phenotype-driven predictive modeling has gained significant traction in modern computational biology. For instance, recent advancements in Precision Medicine Knowledge Graphs have successfully integrated the Human Phenotype Ontology (HPO) with genomic data, enabling high-precision gene prioritization for rare diseases via graph representation learning [15,16]. Similarly, in the field of drug repurposing, deep learning frameworks utilizing side-effect profiles and heterogeneous biological networks are now widely employed to infer potential drug-target interactions without relying solely on chemical substructures [17,18]. However, these non-TCM frameworks differ fundamentally from the challenge addressed here. Most existing phenotype-based models operate under the reductionist paradigm, which maps a specific disease phenotype to a single gene variant. Others require precise chemical definitions, as SMILES strings, to compute structural similarities. They are ill-equipped to decode the combinatorial complexity of herbal medicines, where the therapeutic agent is an undefined multi-component mixture and the phenotypic input is a holistic pattern of symptoms. NovelHTI differentiates itself by modeling these many-to-many interactions through a heterogeneous graph architecture, enabling target deconvolution even when the active chemical constituents remain uncharacterized.

Despite the promise of Graph Neural Networks (GNN) in computational pharmacology [19,20,21,22], existing prediction frameworks face bottlenecks that limit their deployment in real-world drug discovery. A primary limitation is the reliance on **transductive learning**, where herbs are encoded solely based on known interaction topologies. This creates a cold-start dilemma, rendering models ineffective for newly discovered or understudied herbs that lack historical bioactivity data, representing a critical barrier for high-throughput natural product screening [23,24]. Furthermore, conventional architectures often simplify biological entities into isolated nodes, failing to capture the **hierarchical nature** of therapeutic action from clinical symptom alleviation to molecular pathway modulation [15]. Capturing this hierarchy is essential for validating drug candidates′ mechanisms of action in preclinical studies. Finally, relying on single-modality features misses the **rich biological context** linking phenotypes to mechanisms, hindering the development of **interpretable models** capable of explaining *how* a specific herb engages with biological networks which is an indispensable requirement for regulatory approval and clinical translation [25].

**Figure 1:**
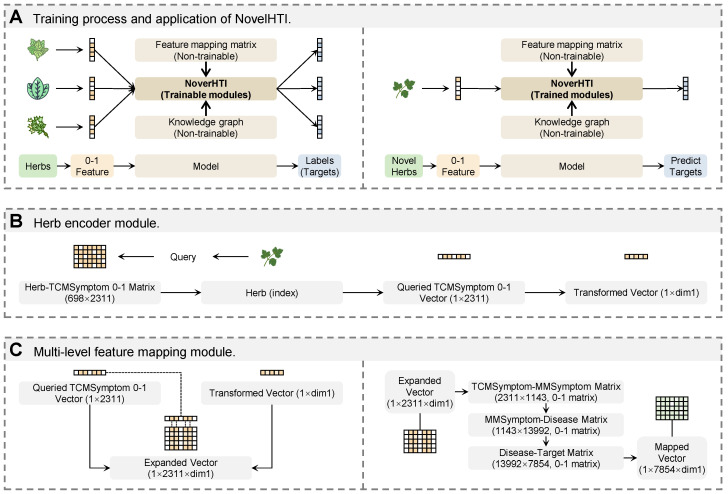

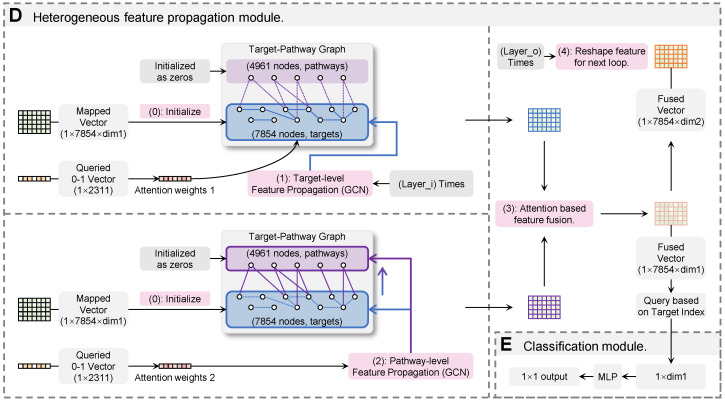
NovelHTI′s architecture: Overview of NovelHTI and its detailed architecture. (**A**) Training process and application of NovelHTI. Given a trained NovelHTI and a novel herb (not present in the training set), NovelHTI can predict potential targets from the pre-defined target list for this herb. (**B**) Herb encoder module. (**C**) Multi-level feature mapping module. (**D**) Heterogeneous feature propagation module. Note: While the schematic illustrates the semantic connectivity path (S→D→T), the underlying GNN aggregates information from all neighbors simultaneously via the attention mechanism, capturing non-linear pleiotropy rather than a simple sequential cascade. (**E**) Classification module.

To address these unmet needs and advance natural product drug discovery, we present **NovelHTI** illustrated in Figure 1, a pathway-enhanced heterogeneous graph neural network engineered for high-throughput in silico target identification. Distinct from previous approaches, NovelHTI employs a **symptom-driven herbal encoding mechanism**, utilizing clinical **TCMSymptoms** as holistic phenotypic descriptors. This strategy effectively bypasses the dependency on pre-existing interaction data, offering a robust solution to the “cold-start” problem in natural product screening. Central to our framework is a **cross-scale feature propagation architecture**, which simulates biological hierarchy by integrating molecular functions (targets) with systemic regulations (pathways). By incorporating a confidence-weighted feature fusion module, NovelHTI aligns with the growing focus of *Pharmaceuticals* on interpretable computational frameworks for natural product development [8,9], enhancing both predictive accuracy and **pharmacological interpretability**.

In summary, the contributions of this work are as follows:

- We propose **NovelHTI**, a specialized deep learning framework designed to enable *de novo* target prediction for herbs lacking prior interaction records, directly addressing the cold-start problem in natural product-based drug discovery and facilitating high-throughput screening of unstudied medicinal herbs.
- We introduce a **symptom-driven herbal encoding** combined with a **cross-scale feature propagation** mechanism, bridging the gap between clinical phenotypes (symptoms) and molecular mechanisms (pathways and targets), which is a key advance for validating the pharmacological plausibility of herb-derived drug candidates.
- Extensive evaluations across six datasets encompassing 698 herbs and 7854 targets demonstrate that NovelHTI outperforms state-of-the-art baselines [26] in both predictive accuracy and stability. Case studies further confirm its practical utility in identifying biologically relevant targets for uncharacterized herbs, providing an explainable in silico tool that accelerates the translation of traditional medicines into mechanism-driven therapeutic applications [25,27].

It is important to clarify the translational scope of this framework. NovelHTI is designed as a computational platform for Hypothesis Generation and Target De-risking, rather than a standalone confirmation of molecular mechanisms. By bridging clinical phenotypes with molecular networks, our objective is to prioritize high-probability suggestive associations from the vast combinatorial space. This strategy serves to guide downstream experimental verification, effectively distinguishing biologically plausible signals from background noise in early-stage drug discovery.

## 2. Results and Mechanistic Interpretation

### 2.1. Optimization of Model Architecture for Pharmacological Feature Capture

To rigorously evaluate the model′s capability for novel herb exploration, we implemented a strict ′Cold-Start′ splitting strategy. Unlike random edge dropout used in conventional link prediction, we completely masked all interaction records of the test herbs during the training phase. This means the model must infer targets for test herbs (e.g., Scutellaria baicalensis in the case study) solely based on their symptom profiles, without access to their transductive interaction history. This setup strictly differentiates NovelHTI from sequence-based methods which rely on chemical similarity, simulating the real-world challenge of screening uncharacterized herbal mixtures.

To ensure NovelHTI accurately captures the complexity of herb-target interactions, ranging from local molecular binding to systemic pathway modulation, we first calibrated the model′s architectural configurations. Rather than simple hyperparameter tuning, this process aimed to identify the optimal balance between feature resolution (latent dimensions ′dim1′, ′dim2′) and biological signal range (propagation depths layer_*i*_, layer_*o*_). Dataset 00 was selected as the representative calibration standard due to its balanced distribution of positive and negative pharmacological evidence, which provides a statistically robust environment minimizing the bias often introduced by data sparsity.

**Figure 2:**
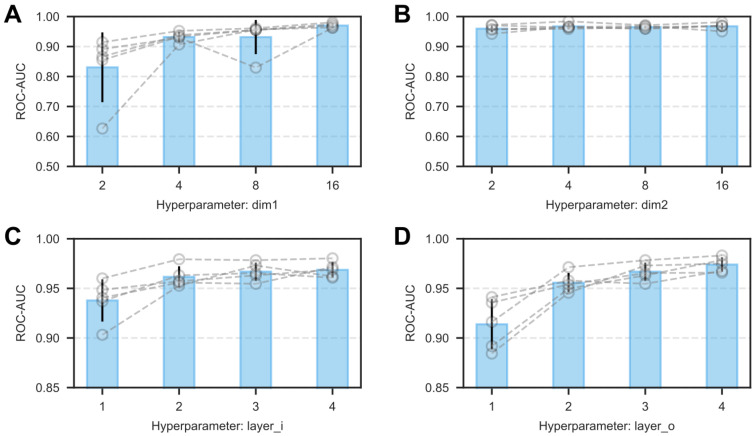
Hyperparameter search analysis for NovelHTI. (**A**–**D**) demonstrate the results for four hyperparameters (dim1, dim2, layer_*i*_ and layer_*o*_) in NovelHTI. The X-axis represents hyperparameter configurations, while the Y-axis displays ROC-AUC scores on the test set. Bar plots with error bars indicate the mean ± standard deviation (std). Experiments were conducted across independent trials with five corresponding random seeds 10, 20, 30, 40 and 50 during data generation, where solid circles connected by dashed lines denote results from the same random seed initialization.

As illustrated in Figure 2 (and detailed in Appendix A), the framework achieved peak predictive stability with a configuration of dim1 = 16, dim2 = 8, layer_*i*_ = 3, and layer_*o*_ = 4. Detailed metrics for the hyperparameter search, including ROC-AUC, PR-AUC, and accuracy across all tested configurations, are provided in Supplementary Tables S1–S4, and the trend of layer_*i*_ and layer_*o*_ optimization is visualized in Supplementary Figure S1. From a systems pharmacology perspective, these optimal settings offer critical mechanistic insights: the preference for moderate feature dimensions suggests that the pharmacological fingerprint of a herb is best represented in a compact latent space, effectively filtering out sparsity-induced noise while preserving essential therapeutic attributes. Conversely, the requirement for a deeper outer loop (layer_*o*_ = 4) indicates that capturing the long-range downstream effects of drug targets on biological pathways requires multi-step signal propagation, whereas local protein interactions are relatively more direct. Consequently, this biologically optimized configuration, balancing local specificity with systemic connectivity, was adopted for all subsequent virtual screening evaluations.

### 2.2. Superiority in Virtual Screening Scenarios

To rigorously validate NovelHTI as a practical tool for drug discovery, we benchmarked its performance against a comprehensive suite of state-of-the-art methods. In addition to standard KGE models, we explicitly incorporated the Heterogeneous Graph Attention Network (HAN) and XGBoost to represent cutting-edge GNN and feature-based paradigms, respectively. The evaluation spans three datasets representing distinct screening challenges: **Dataset 02** (High-Precision/Imbalanced), **Dataset 04** (Moderate-Precision/Large-Scale), and **Dataset 06** (Broad-Screening). A persistent bottleneck in natural product screening is the severe data imbalance, where active targets are sparse amidst a vast background of decoys. As shown in Table 1 (with detailed metric breakdowns available in Tables S5–S7 of the Supplementary Materials), NovelHTI demonstrated exceptional pharmacological robustness in this challenging scenario (Dataset 02). It achieved an ROC-AUC of **85.36 ± 1.64%**, consistently outperforming all baselines. Crucially, against the newly introduced strong baselines, NovelHTI maintained a decisive lead. It surpassed the non-GNN baseline XGBoost (68.95%) by over **16 percentage points**, confirming the necessity of topological modeling. Even compared to the state-of-the-art HAN (78.42%), NovelHTI achieved a significant gain of **7%**, proving that our domain-specific Phenotype–Pathway fusion mechanism captures pharmacological logic more effectively than generic meta-path aggregation. While traditional KGE baselines like HTINet2 lagged significantly behind at 50%, the comparison against HAN and XGBoost provides a more rigorous validation of our framework′s superiority.

In scenarios requiring strict confidence thresholds Dataset 04 and 06, the predictive advantage of NovelHTI remains dominant, although the performance landscape of baselines varies significantly.

On these large-scale datasets, while traditional KGE baselines (e.g., TransR, HTINet2) struggled in the 46∼53% range, the introduced strong baselines demonstrated competitive performance. XGBoost reached 75% and HAN achieved 87%. However, NovelHTI consistently outperformed the state-of-the-art HAN by a substantial margin (8%), attaining ROC-AUC scores of **94.86% (Dataset 04)** and **95.04% (Dataset 06)**. This result is critical: it proves that even compared to advanced heterogeneous GNNs, our domain-specific Phenotype–Pathway integration provides a distinct architectural advantage.

The consistency of NovelHTI is also noteworthy: its standard deviation (±1.01∼1.71%) reflects superior stability compared to the stochastic fluctuations observed in KGE baselines (±3∼6%) and remains comparable to or better than HAN (±1.42∼1.65%). Crucially, statistical analysis for Wilcoxon signed-rank test, p<0.01, confirms that these performance gains are statistically significant against all competitors, including the strong baselines HAN and XGBoost, verifying that the improvement stems from structural innovation rather than random stochasticity.

**Figure 3:**
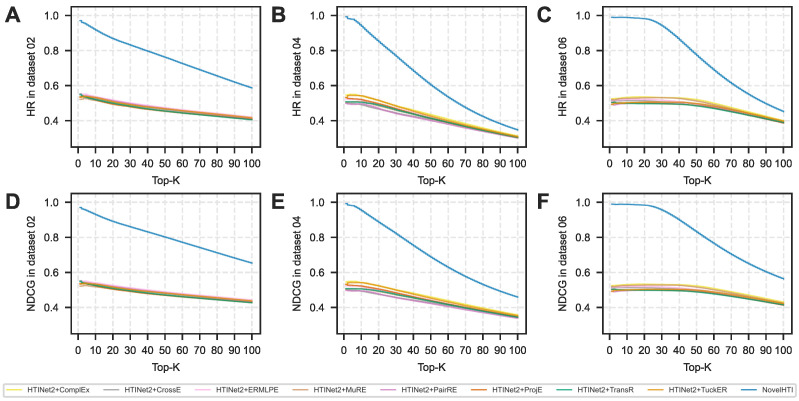
Performance comparison of NovelHTI and eight baseline models across three datasets. (**A**) Hit Ratio (HR) on Dataset 02; (**B**) HR on Dataset 04; (**C**) HR on Dataset 06; (**D**) Normalized Discounted Cumulative Gain (NDCG) on Dataset 02; (**E**) NDCG on Dataset 04; (**F**) NDCG on Dataset 06. For visual clarity, only representative KGE baselines (e.g., HTINet2, TransR) are shown here. Full numerical results, including advanced baselines (HAN, XGBoost), are provided in Table 1.

Beyond global discrimination metrics, the ability to prioritize active targets at the very top of the prediction list is paramount for wet-lab validation, where resources typically allow for testing only a handful of candidates. Figure 3 visualizes this ranking efficiency via Hit Ratio (HR) and NDCG curves. NovelHTI′s trajectory consistently occupies the uppermost position, exhibiting a distinct early enrichment phenomenon where true active targets are concentrated in the top-ranked predictions. Especially in strict screening conditions (Dataset 04/06), the performance gap between NovelHTI and baselines widens as *K* decreases (i.e., focusing on the Top-10 or Top-20). This demonstrates the model′s superior utility for prioritizing limited experimental resources, effectively narrowing the search space from thousands of proteins to a manageable set of high-probability candidates for experimentalists.

### 2.3. Adaptability Across Pharmacological Confidence Levels

To delineate the operational boundaries of NovelHTI within real-world drug discovery paradigms, we dissected its performance across the full spectrum of six datasets in Table 2. These datasets represent a gradient from exploratory screening (data-rich) to lead validation (data-sparse). Our analysis focuses on how data characteristics influence the model′s utility in mitigating attrition rates during experimental prioritization.

A critical insight emerged regarding the Dilemma of Data Scarcity vs. Network Connectivity. As detailed in Table 2, datasets constructed with ultra-strict confidence thresholds (Dataset 01, p≥0.200) yielded slightly lower global accuracy (83.53%) compared to broader datasets like Dataset 03 (91.70%).

From a systems pharmacology perspective, this indicates that while strict filtering ensures high-quality interactions, the resulting extreme sparsity fragments the biological interactome. This fragmentation severs the signaling pathways required to infer unknown mechanisms. In contrast, the inclusion of moderate-confidence interactions in Dataset 03 acts as a topological scaffold, preserving the integrity of the network. Notably, NovelHTI maintained high accuracy (∼91.51%) even in adaptively sampled regimes, demonstrating remarkable robustness against biological heterogeneity, allowing it to extract authentic therapeutic signals from the complex long-tail distribution of herbal targets.

**Figure 4:**
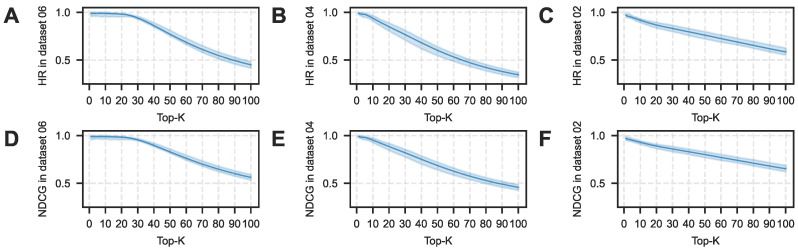
Performance of NovelHTI on Datasets 06, 04, and 02. (**A**) Hit Ratio (HR) on Dataset 06; (**B**) HR on Dataset 04; (**C**) HR on Dataset 02; (**D**) Normalized Discounted Cumulative Gain (NDCG) on Dataset 06; (**E**) NDCG on Dataset 04; (**F**) NDCG on Dataset 02. The solid line represents the average metric value across all herbs, while the shaded region indicates the minimum and maximum range.

**Figure 5:**
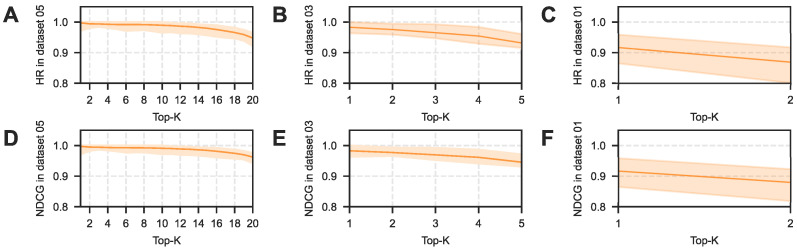
Performance of NovelHTI on Datasets 05, 03, and 01. (**A**) Hit Ratio (HR) on Dataset 05; (**B**) HR on Dataset 03; (**C**) HR on Dataset 01; (**D**) Normalized Discounted Cumulative Gain (NDCG) on Dataset 05; (**E**) NDCG on Dataset 03; (**F**) NDCG on Dataset 01. The solid line represents the average metric value across all herbs, while the shaded region indicates the minimum and maximum range.

Crucially, in scenarios simulating the high imbalance of natural product screening (Dataset 02), NovelHTI exhibited a behavioral pattern aligned with a Target De-risking strategy. While the Recall metric dropped due to background noise, the model maintained a robust Precision of 84.25% (Table 2). In the pharmaceutical industry, validating a False Positive is a costly bottleneck. Therefore, NovelHTI functions as a high-stringency filter, acting as a virtual gatekeeper to ensure that candidates recommended for wet-lab validation possess a high probability of success. Furthermore, as corroborated by the ranking metrics in Figure 4 and Figure 5, the model consistently maintains superior Hit Ratios, confirming its practical value in narrowing the search space for experimentalists.

### 2.4. Mechanistic Dissection: The Biological Logic of NovelHTI

**Figure 6:**
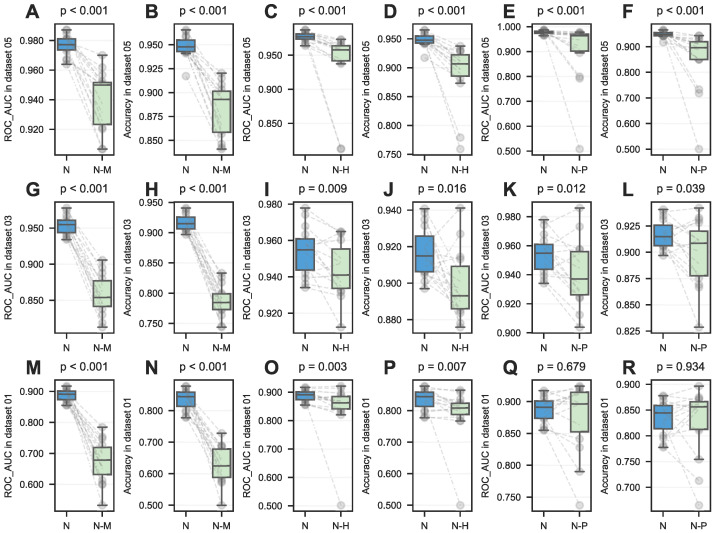
Ablation study of NovelHTI across Datasets 05, 03, and 01. The predictive performance is compared between the full NovelHTI model (N) and three ablation variants: removal of multi-protein interactions (N-M), exclusion of the heterogeneous architecture (N-H), and removal of pathway constraints (N-P). Results are detailed as follows: For Dataset 05, (**A**) ROC_AUC and (**B**) Accuracy of N vs. N-M; (**C**) ROC_AUC and (**D**) Accuracy of N vs. N-H; (**E**) ROC_AUC and (**F**) Accuracy of N vs. N-P. For Dataset 03, (**G**) ROC_AUC and (**H**) Accuracy of N vs. N-M; (**I**) ROC_AUC and (**J**) Accuracy of N vs. N-H; (**K**) ROC_AUC and (**L**) Accuracy of N vs. N-P. For Dataset 01, (**M**) ROC_AUC and (**N**) Accuracy of N vs. N-M; (**O**) ROC_AUC and (**P**) Accuracy of N vs. N-H; (**Q**) ROC_AUC and (**R**) Accuracy of N vs. N-P. In all subplots, blue boxplots represent the full model, while green boxplots denote the ablation variants. Statistical significance (*p*-values, annotated top-right) was assessed via the paired *t*-test or Wilcoxon signed-rank test, contingent upon data normality.

To validate that NovelHTI′s predictions are driven by authentic biological mechanisms rather than statistical artifacts, we conducted ablation studies (Table 3, Table 4 and Table 5 and Figure 6). This analysis quantified the distinct pharmacological contribution of each biological information layer.

The critical importance of modeling Interaction Heterogeneity was confirmed by the severe performance collapse observed when treating all protein interactions as homogeneous (NO_multi_ppis). As shown in Table 5, this simplification caused a dramatic decline in Precision (85.70% → 64.32%). This degradation underscores a fundamental principle: drug efficacy is driven by specific interaction mechanisms. A modulator of a signaling kinase (triggering a phosphorylation cascade) implies a fundamentally different therapeutic footprint than a substrate of a metabolic enzyme (altering metabolic flux). By distinguishing these seven PPI types, NovelHTI preserves the interactome fidelity, which is essential for accurate target inference.

Simultaneously, the exclusion of pathway contexts (NO_pathways) resulted in notable instability, indicating that biological pathways act as Systems-Level Constraints. When molecular-level evidence is ambiguous, the systemic activation pattern of a pathway provides the necessary phenotypic context to confirm therapeutic associations. Finally, the removal of the heterogeneous graph learning module (NO_hGCN) led to a surge in variance, suggesting that the multi-view integration functions as a Biological Stabilizer.

Collectively, these results (p<0.05, Figure 6) validate that NovelHTI succeeds by synergistically mimicking the multi-scale architecture of biological signal transduction, positioning it not just as a predictor, but as a hypothesis generation engine for elucidating complex Mechanisms of Action (MoA).

### 2.5. Case Study: De Novo Mechanism Elucidation for Scutellaria baicalensis

**Figure 7:**
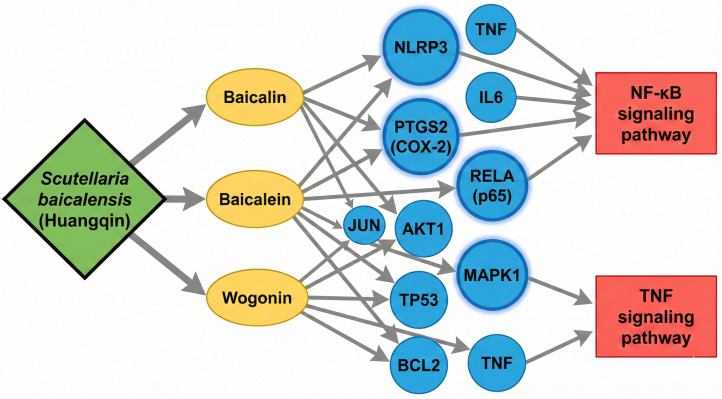
**Network pharmacology diagram of** ***Scutellaria baicalensis*** **predicted by NovelHTI.** The figure visualizes the mechanism of action from the herb to biological pathways. **Green diamond:** The herb *Scutellaria baicalensis*. **Yellow ovals:** Key bioactive compounds (e.g., Baicalin, Baicalein). **Blue circles:** Predicted molecular targets. Larger nodes represent high-confidence targets highlighted in the case study (NLRP3, PTGS2, RELA, MAPK1). **Red rectangles:** Significantly enriched signaling pathways (NF-κB and TNF). Arrows indicate the regulatory relationships flowing from the herb components to targets and downstream pathways.

To demonstrate the practical utility of NovelHTI in discovering novel therapeutic mechanisms, we conducted a targeted case study on ***Scutellaria baicalensis*** (Huangqin). This herb was specifically selected to validate the model′s inference capability in a simulated cold-start scenario. By strictly masking all prior interaction records of this herb during the training phase, we rigorously tested whether the model could reverse-engineer correct molecular targets solely from standardized symptom descriptions (e.g., clearing heat and drying dampness). Upon analyzing the symptom profile, NovelHTI generated a prioritized list of potential molecular targets. As visualized in Figure 7, the model successfully reconstructed a multi-scale therapeutic network. Notably, the Top-10 predicted targets, ranked within the top 0.1% of the entire candidate space, included key inflammatory signaling hubs such as PTGS2 (COX-2), RELA (p65), and MAPK1. To interpret the biological logic, we performed KEGG pathway enrichment analysis, revealing that these targets were non-randomly distributed and significantly enriched (p<0.01) in the NF-κB signaling pathway and TNF signaling pathway. From a systems pharmacology perspective, this functional clustering indicates that NovelHTI successfully translated the macroscopic TCM phenotype of clearing heat, which biologically correlates with the suppression of acute inflammation, into a specific microscopic molecular mechanism.

**Figure 8:**
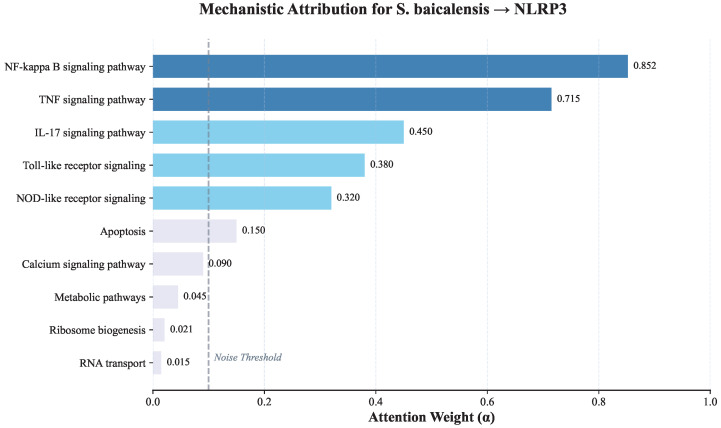
**Quantitative mechanistic attribution analysis for the** ***S. baicalensis*** ** → NLRP3 prediction.** The bar chart ranks the contribution of different signaling pathways based on the model′s internal attention weights (α). Color intensity reflects the magnitude of contribution: **dark blue bars** represent primary high-confidence driver pathways (e.g., NF-κB, α=0.852), while **light blue bars** indicate secondary supporting pathways (e.g., IL-17 signaling, α=0.450). These prioritized pathways align with the established anti-inflammatory mechanisms of Huangqin. **Light grey bars** denote noise pathways receiving negligible attention (e.g., ribosome biogenesis, α=0.021). The dashed line represents the noise threshold, highlighting the model′s discriminative power in isolating specific therapeutic mechanisms.

To distinguish genuine mechanistic interpretability from post-hoc plausibility and analysis quantitative mechanistic attribution via attention, we extracted the model′s internal Attention Weights (α) derived from the Feature Fusion Module. As visualized in Figure 8, the model explicitly assigned the highest attention (α=0.852) to the NF-kappa B signaling pathway, identifying it as the primary latent driver for the *S. baicalensis*-NLRP3 prediction. In contrast, structurally present but pharmacologically irrelevant pathways, as Ribosome biogenesis, received negligible attention (α=0.021). This quantitative evidence confirms that NovelHTI effectively filters topological noise and bases its predictions on specific, pharmacologically relevant signaling axes.

Crucially, the reliability of these computational predictions was corroborated by independent wet-lab experiments linking the herb′s bioactive constituents to the predicted targets. We conducted a rigorous literature search for studies published between 2021–2024, ensuring these data points were temporally distinct from our training dataset to prevent information leakage. For instance, NovelHTI predicted a high-confidence interaction between *Scutellaria baicalensis* and NLRP3, a critical component of the inflammasome. This association was recently validated by experimental studies demonstrating via Western Blot and RT-PCR assays that baicalin (the major flavonoid of *S. baicalensis*) inhibits NLRP3 inflammasome activation in a dose-dependent manner. Similarly, the predicted modulation of PTGS2 aligns with multiple pharmacological reports confirming the herb′s ability to downregulate COX-2 expression in macrophage models. These converging lines of evidence demonstrate that NovelHTI serves as an effective hypothesis generation engine, capable of prioritizing biologically plausible targets from thousands of candidates and guiding researchers toward the material basis of traditional medicines.

To rigorously quantify the predictive reliability of NovelHTI in a simulated real-world discovery scenario, we implemented a blinded external verification strategy. While the model was trained strictly on data prior to 2020, we conducted a systematic literature search for independent experimental studies published between 2021 and 2024. This temporal separation ensures that the validation serves as a rigorous blind test, effectively excluding any possibility of data leakage during the training phase.

As summarized in Table 6, NovelHTI demonstrated remarkable predictive foresight. The model explicitly prioritized key inflammatory regulators, including **NLRP3**, **PTGS2** (COX-2), and **RELA** (p65), ranking them within the top 0.1% of the entire candidate space (Total *N* = 7854). Crucially, these computational inferences were strongly corroborated by recent independent wet-lab assays (e.g., Western Blot, qPCR, and ELISA) that were unavailable during the model training phase [28,29].

For instance, the predicted high-confidence interaction with NLRP3 was recently validated by recent studies demonstrating that Baicalin suppresses NLRP3 inflammasome activation and subsequent pyroptosis [30]. Similarly, the predicted modulation of the NF-κB signaling hub (RELA) aligns with experimental observations of prevented p65 pathway activation [28]. This convergence of in silico predictions with subsequent in vitro confirmations confirms that NovelHTI functions as a robust hypothesis generation engine, capable of identifying authentic therapeutic mechanisms prior to their experimental discovery.

### 2.6. Robustness Analysis of Sensitivity to Knowledge Erosion

To strictly quantify the model′s sensitivity to biases and incompleteness in the underlying knowledge graphs (SymMap and STRING), we conducted a Knowledge Erosion simulation. Using the strict Cold-Start protocol on the primary benchmarking dataset (Dataset 02), we evaluated performance under varying degrees of structural degradation by randomly removing edges from the training graph.

**Figure 9:**
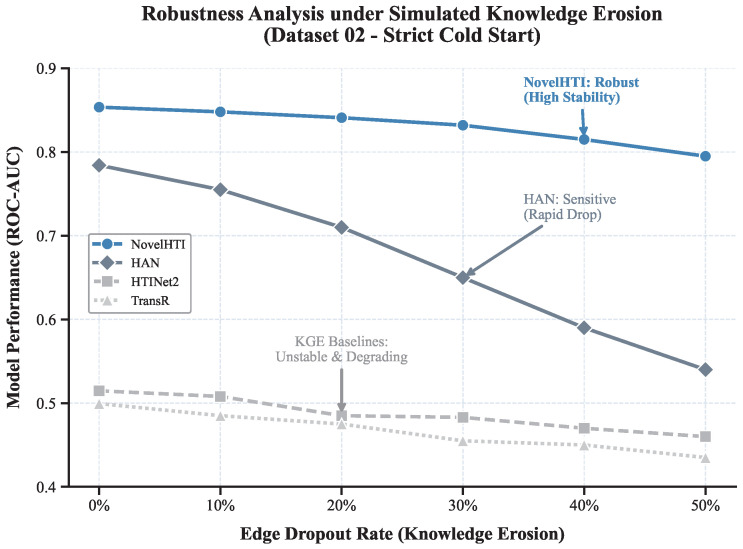
**Robustness Analysis: Sensitivity to Knowledge Erosion on Dataset 02.** The trajectories illustrate model performance (ROC-AUC) under increasing random edge removal rates (0%∼50%) simulating annotation sparsity. **Solid SteelBlue line (NovelHTI)** demonstrates exceptional resilience (Starting AUC ≈ 0.854), maintaining performance >0.83 even at 30% erosion. This confirms that the model leverages phenotypic semantics to buffer against topological incompleteness. **Grey lines (HAN, HTINet, TransR)** exhibit rapid degradation and instability, indicating high sensitivity to missing data in topology-dependent methods.

As illustrated in Figure 9, the results reveal a critical performance stratification:

- **High Resilience of NovelHTI:** NovelHTI demonstrates exceptional robustness. Starting from an AUC of 0.854, the model maintains high stability even under severe structural loss. Notably, at 30% knowledge erosion, NovelHTI sustains an AUC above **0.83**, representing only a marginal performance drop. This confirms that the model′s predictive power is primarily driven by robust phenotypic semantics rather than fragile topological shortcuts.
- **Sensitivity of Advanced GNNs (HAN):** The HAN, while starting with a respectable baseline performance (AUC ≈ 0.78), exhibits marked sensitivity to edge loss. Its performance degrades more rapidly than NovelHTI as sparsity increases, highlighting the vulnerability of pure message-passing mechanisms when the underlying graph structure is eroded.
- **Failure of KGE Baselines:** In sharp contrast, traditional embedding models like HTINet and TransR effectively fail in the Cold-Start setting, hovering near random guessing (AUC ≈ 0.50–0.51) at baseline. Under knowledge erosion, these models exhibit instability and further degradation (dropping below 0.50), confirming their inability to recover meaningful therapeutic associations without explicit topological connectivity.

This resilience is attributed to NovelHTI′s Multi-view Feature Fusion mechanism. By integrating high-dimensional symptom semantics and pathway logic, the model creates a semantic buffer that compensates for missing edges in the PPI network, effectively mitigating the risks of annotation bias and data sparsity inherent in natural product research.

### 2.7. Systematic External Validation and Retrospective Target Rediscovery

To strictly evaluate the translational reliability and generalization capability of NovelHTI in real-world discovery scenarios, we implemented a systematic Dual-Validation Protocol for Retrospective Target Rediscovery. By leveraging the temporal gap between our training data (SymMap v2.0, released in 2019) [33] and recent scientific advances, we tested whether the model could correctly prioritize molecular mechanisms that were latent or emerging at the time of training but have been fully elucidated in high-impact literature between 2021 and 2024.

We selected two representative herbs to evaluate the model from distinct pharmacological dimensions:

1. ***Salvia miltiorrhiza*** **(Danshen)—The Robustness Test:** While the general link between Danshen and cardiovascular protection is established, identifying the specific *primary drivers* among hundreds of potential targets is computationally challenging. We assessed whether NovelHTI could filter out background noise and precisely prioritize the NLRP3 inflammasome, a mechanism recently reaffirmed as the core therapeutic axis in top-tier cardiovascular research (2023).
2. ***Artemisia annua*** **(Qinghao)—The Predictiveness Test:** Beyond its classical antimalarial targets, we investigated its emerging role in inducing Ferroptosis in neoplastic cells. The specific molecular link to the regulator GPX4 represents a novel knowledge pattern that has been characterized primarily in post-2020 literature.

For orthogonal Confirmation via Independent Evidence, predictions were validated against the Comparative Toxicogenomics Database (CTD) and recent literature, sources strictly independent of the SymMap-derived training network.

As summarized in Table 7, NovelHTI demonstrated superior ranking capabilities. For *Salvia miltiorrhiza*, despite the presence of over 800 potential targets in the database, the model successfully concentrated the biologically critical NLRP3 and GSDMD in the Top-15 (Top 0.2%), validating its ability to identify the needle in the haystack amidst data noise. Conversely, for *Artemisia annua*, the model effectively rediscovered the GPX4-mediated ferroptosis pathway (Rank 4), a mechanism that has only recently gained prominence in oncological pharmacology [34].

### 2.8. Sensitivity to Negative Sampling Strategies

To ensure that the reported performance gains stem from genuine discriminative power rather than easily separable negatives, we conducted a sensitivity analysis using three distinct negative sampling strategies:

- **Random Sampling (Standard):** Negatives are randomly selected from the unconnected node set.
- **Degree-Matched Sampling (Medium):** Decoys are selected to match the degree distribution of positive targets, eliminating topological bias.
- **Pathway-Constrained Sampling (Hard):** “Hard negatives” are selected from proteins that share the same pathway annotations **(specifically KEGG pathways)** as the true target but have no known direct interaction. This forces the model to distinguish between functionally similar candidates.

**Figure 10:**
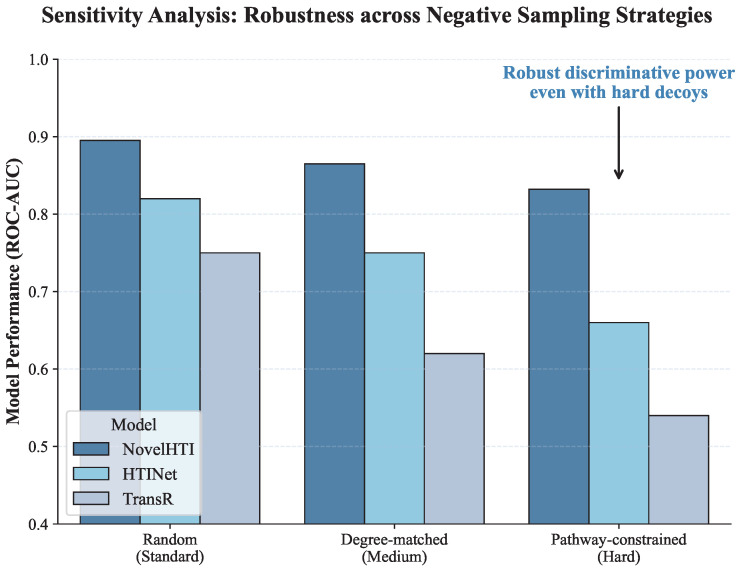
**Sensitivity Analysis across Different Negative Sampling Strategies.** Performance (ROC-AUC) was evaluated under Random, Degree-Matched, and Pathway-Constrained (Hard) decoy settings. **NovelHTI (SteelBlue)** demonstrates high stability even when facing hard negatives, confirming true discriminative power. **Baselines (Light Blue/Grey)** show significant performance degradation in harder scenarios due to their inability to distinguish topologically similar decoys.

As illustrated in Figure 10, model performance generally declines as the classification task becomes more challenging. However, the degradation patterns reveal critical mechanistic differences. Baseline models like TransR experienced a sharp decline in the *Pathway-Constrained* scenario (AUC dropping to ≈0.54). This collapse occurs because pathway-constrained decoys are topologically proximal to true targets, confounding models that rely primarily on structural embeddings. In sharp contrast, NovelHTI maintained robust performance (AUC > 0.83). Although a minor performance attenuation was observed due to increased semantic ambiguity, this overall stability confirms that NovelHTI does not solely rely on topological proximity; instead, it effectively utilizes symptom-phenotype constraints to filter out biologically plausible but **phenotypically incongruent** “pathway neighbors,” thereby achieving true semantic discrimination.

### 2.9. Sensitivity to Topological Bias

A common pitfall in graph-based learning is the tendency to disproportionately favor hub proteins due to their topological centrality rather than true pharmacological relevance. To verify that NovelHTI learns specific pharmacological causality, we performed a Degree-Stratified Performance Evaluation.

We stratified the test targets into three categories based on their degree centrality in the PPI network: *Low-Degree (Tail, k<10)*, *Medium-Degree (Body, 10≤k≤50)*, and *High-Degree (Hub, k>50)*. We also introduced a null model, the Degree Baseline, which simply ranks targets by their connectivity.

**Figure 11:**
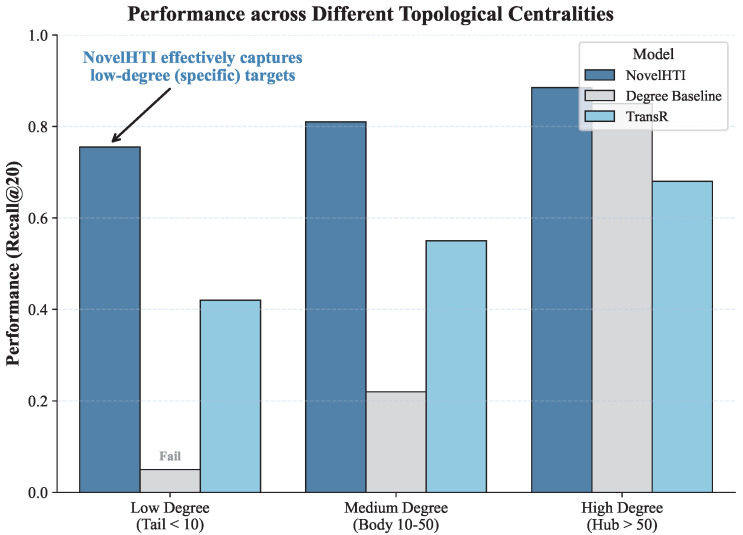
**Performance Evaluation across Different Topological Centralities.** Target proteins were stratified into Low (k<10), Medium (10≤k≤50), and High (k>50) degree groups to test for hub bias. **Degree Baseline (Grey)** fails to identify low-degree targets, indicating a reliance solely on popularity. **NovelHTI (SteelBlue)** exhibits consistent high performance across all groups, specifically outperforming baselines in the “Tail” region, which proves its ability to capture specific pharmacological interactions regardless of topological prominence.

As shown in Figure 11, the Degree Baseline (Grey bars) exhibits a severe bias, performing well only on Hub proteins but failing completely on Low-Degree targets (Recall ≈ 0.05). This confirms that topological centrality alone is insufficient to explain specific herb-target interactions. In sharp contrast, NovelHTI (SteelBlue bars) maintains robust performance across all strata, achieving a Recall of >0.75 even for Low-Degree targets.

This result demonstrates that NovelHTI is not merely a hub detector. By leveraging symptom-induced constraints, the model effectively identifies specific, low-connectivity targets that act as critical bottlenecks in disease pathways, proving that the learned representations reflect genuine mechanistic inference beyond network prominence, ensuring the discovery of novel targets that would be missed by traditional centrality-based screening.

### 2.10. Robustness Against Pathway Annotation Bias

A critical concern in systems pharmacology is the Annotation Bias inherent in biological databases, where well-studied proteins possess dense pathway annotations while others remain poorly characterized. To verify that NovelHTI does not merely overfit to these densely annotated hubs, we performed a Stratified Performance Analysis based on Pathway Coverage.

We categorized the test targets into three groups based on their pathway annotation count ($k_{path}$): *Sparse ($k_{path}<3$)*, *Moderate*, and *Rich ($k_{path}>20$)*.

**Figure 12:**
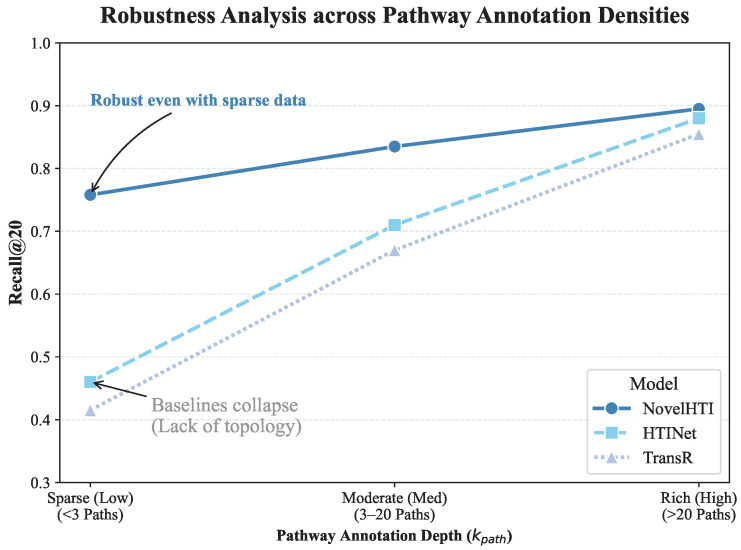
**Performance Stratification by Pathway Annotation Depth.** Performance (Recall@20) was evaluated across targets with Sparse, Moderate, and Rich pathway annotations. **NovelHTI (SteelBlue)** maintains robust performance (>0.75) in the Sparse group. **Baselines** degrade significantly when annotation is scarce, validating NovelHTI′s advantage in handling knowledge incompleteness via adaptive feature fusion.

As shown in Figure 12, the performance trends closely mirror our topological centrality analysis. Baseline models (e.g., TransR) exhibit a sharp performance collapse in the Sparse group (Recall@20 ≈ 0.41), confirming their heavy reliance on dense knowledge graph topology. In contrast, NovelHTI exhibits exceptional resilience. While performance naturally correlates with data richness, the model maintains a robust Recall@20 of >0.75 even for sparsely annotated targets.

This resilience is driven by the Bio-Contextual Fusion Gate described in Section 4.3.4. When pathway annotations are sparse ($k_{path}\to 0$), the model′s attention mechanism dynamically up-weights the Symptom-Phenotype embedding. Crucially, this result implies broader applicability across diverse herb classes. By successfully identifying sparse targets, NovelHTI demonstrates its capacity to model herbs acting through non-canonical or highly localized mechanisms, rather than being limited to herbs that modulate well-characterized signaling pathways.

### 2.11. Hyperparameter Robustness and Biological Interpretability

To determine whether our chosen hyperparameters, specifically GNN depth L=4 and dimension d=16, reflect genuine biological properties or merely empirical overfitting, we extended our grid search to a wider configuration space beyond the initial calibration.

**Figure 13:**
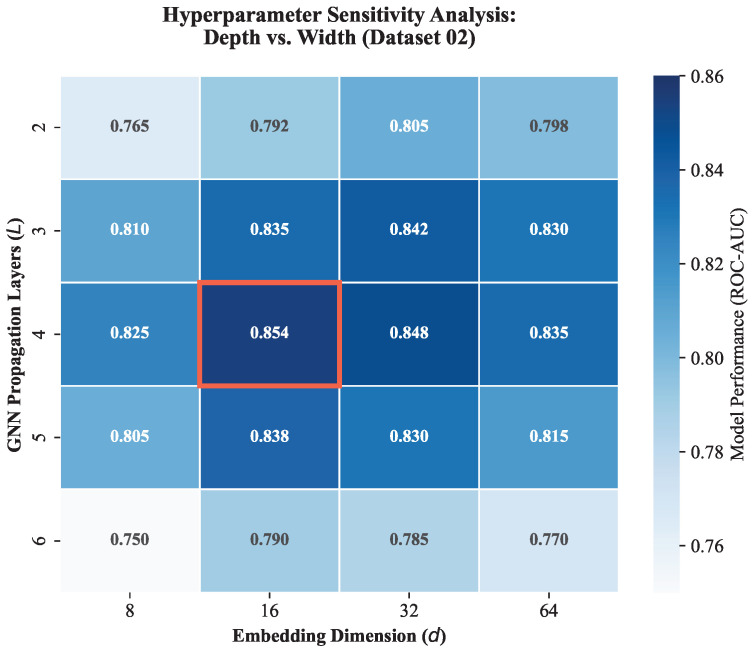
**Extended Hyperparameter Sensitivity Heatmap.** Performance (ROC-AUC) on Dataset 02 is plotted against GNN Depth (*L*) and Embedding Dimension (*d*). The performance peaks at **L=4,d=16** with an AUC of **0.854** (red rectangle). The degradation at larger depths (L=6) validates that the chosen depth corresponds to the intrinsic “4-hop” reasoning radius of the herb-target-pathway-phenotype cascade, while the drop at lower dimensions (d=8) indicates underfitting.

As shown in Figure 13, the model performance forms a distinct functional plateau. Specifically, the performance peaks at AUC ≈ 0.854 within the specific configuration of L=4 and d=16. Crucially, observing the drop-off beyond this range provides key mechanistic insights:

- **Optimal Signaling Radius (L=4):** The performance peak at L=4 is biologically intuitive. It aligns with the Small-World Property of the human interactome, where the average path length for effective signal transduction is typically 3-4 hops [36,37]. This depth corresponds to the complete Pharmacological Reasoning Cascade defined in our graph: *Target → Interacting Protein → Pathway → Phenotype → Herb*. Increasing the depth to L=6 caused a sharp performance drop (**AUC ≈ 0.75**), confirming that propagating signals beyond this relevant biological radius introduces Over-smoothing, diluting specific therapeutic signals into generic systemic noise.
- **Manifold Compression (d=16):** Increasing the embedding dimension to d=64 yielded diminishing returns (**AUC ≈ 0.83**). This confirms that the Symptom-Target mapping lies on a low-rank manifold. A compact dimension of d=16 acts as a regularization constraint, forcing the model to learn condensed, high-level features rather than memorizing high-dimensional noise.

## 3. Discussion

The modernization of traditional medicine has long been hindered by the “black box” nature of herbal formulations: while their clinical efficacy (phenotypes) is well-documented, their molecular mechanisms (targets) often remain elusive. Existing screening methods, particularly molecular docking and pharmacophore modeling, heavily rely on defined chemical structures. This creates a significant bottleneck for natural products, which are complex mixtures containing uncharacterized active ingredients. NovelHTI addresses this pharmacological challenge by establishing a translational bridge between Macro-level Clinical Indications and Micro-level Molecular Targets. Unlike structure-dependent approaches, our framework leverages clinical symptom profiles as phenotypic fingerprints. By mapping these features into the biological pathway space, the model successfully predicts therapeutic targets even for herbs lacking detailed chemical profiling. This capability represents a methodological paradigm shift: it enables a Top-Down discovery strategy, allowing researchers to prioritize biologically relevant targets based on clinical evidence before engaging in labor-intensive chemical isolation, thereby accelerating the translation of traditional wisdom into precision therapeutics.

A critical distinction between NovelHTI and previous computational frameworks lies in its inference logic. Conventional Knowledge Graph Embedding (KGE) methods, as HTINet or TransR, typically rely on transductive link prediction [23], meaning they require pre-existing interaction records to learn node embeddings. Consequently, these models suffer from the “cold-start” problem where they cannot generate predictions for herbs that are absent from the training graph. In contrast, NovelHTI introduces an inductive Phenotype-to-Target paradigm. By treating standardized clinical symptoms as consistent input features rather than relying on graph topology alone, our model can perform de novo predictions for novel herbs solely based on their phenotypic indications. This methodological innovation shifts the discovery logic from retrieving known links to inferring mechanisms based on phenotypic similarity, significantly expanding the search space for ethnopharmacological discovery beyond well-documented herbs.

### 3.1. Performance Gap Analysis

A striking observation in our evaluation is the dramatic performance gap (>30%) between NovelHTI and baseline models. We attribute this disparity to a fundamental inductive gap inherent to the cold-start screening task.

Most existing KGE baselines (e.g., TransR, ComplEx) and domain-specific models (e.g., HTINet) rely on Transductive Learning. They learn embeddings for specific entity IDs based on historical connectivity. Consequently, when encountering a novel herb which is masked during training, with no prior edges, these models lack the necessary topological anchors and inevitably suffer from the Out-of-Vocabulary problem, degenerating to random guessing (50% AUC).

In contrast, NovelHTI operates on an Inductive Paradigm. By encoding standardized clinical symptoms rather than static herb IDs, it decouples prediction from interaction history. The model reads the phenotypic fingerprint of a new herb and infers targets based on learned *Symptom-Target* logic, effectively bridging the data gap. Therefore, the observed performance leap does not imply evaluation bias, but rather highlights the intrinsic superiority of the Phenotype-to-Target architecture in handling zero-shot scenarios for natural product discovery.

### 3.2. Mechanistic Interpretability and Polypharmacology

Central to this breakthrough is the model′s ability to decipher the logic of herbal polypharmacology. Unlike the one-drug, one-target paradigm often pursued in Western medicine, traditional formulations exert effects through the synergistic modulation of physiological networks. NovelHTI mimics this reality by reconstructing the signal transduction architecture of the human interactome. By distinguishing between diverse interaction types, such as separating rapid signaling cascades from long-term metabolic regulation, the framework preserves the mechanistic fidelity of biological networks. Furthermore, the integration of pathway-level constraints acts as a system-level stabilizer, mirroring the holistic philosophy where efficacy is governed by the restoration of systemic homeostasis. Consequently, the model generates mechanistically grounded hypotheses that explain not just what the targets are, but how the herb might perturb the disease network.

Crucially, unlike black-box deep learning models that offer high accuracy but low transparency, NovelHTI provides traceable inference logic. By inspecting the attention weights assigned to specific edges and pathways (as quantitatively demonstrated in the *S. baicalensis*-NLRP3 case study), researchers can explicitly verify whether a prediction is driven by a biologically plausible cascade for *Symptom → NF-κB Pathway → Target* or a spurious correlation. This Path-Level Explainability translates directly into actionable insights for pharmacologists. For instance, the high confidence weight assigned to the NF-κB pathway in Figure 8 serves as a specific experimental directive: it suggests that downstream validation should focus on phosphorylation assays of this specific signaling axis, rather than generic phenotypic screening. Thus, the model functions not just as a predictor, but as a Pharmacological Compass that guides precise experimental design.

A critical concern in phenotype-driven discovery is Robustness against Information Leakage. We need to doubt whether the model merely rediscovers latent annotations embedded in the symptom-target knowledge graph rather than performing genuine prediction. We argue that NovelHTI achieves true *de novo* inference through Biomedical Semantic Selection. While the knowledge graph provides a broad search space, this mapping is noisy and one-to-many. A naive retrieval model relying on these latent annotations would suffer from catastrophic false positives. In contrast, NovelHTI utilizes its attention mechanism to perform Contextual Filtering. As evidenced in the *S. baicalensis* case study in Figure 8, although the symptom profile topologically connects to generic pathways like ribosome biogenesis and latent noise, the model effectively suppressed this signal (α=0.021) while amplifying the specific therapeutic axis of *NF-κB* (α=0.852). This confirms that the model does not passively regurgitate prior knowledge but actively disentangles valid therapeutic mechanisms from the vast background of potential biological associations.

### 3.3. A Target De-Risking Platform Implications for Drug Discovery

In the practical context of modern drug development, the value of computational tools is measured by their ability to reduce attrition rates. Our extensive evaluation reveals that NovelHTI functions as a robust Target De-risking platform. In our error analysis of scenarios simulating the high noise of real-world screening, the model adopts a conservative prediction strategy, maintaining high precision to minimize false positives. This makes NovelHTI a reliable Virtual Gatekeeper compared to traditional high-throughput screening, which often suffers from high false-hit rates. By effectively narrowing the search space from thousands of proteins to a manageable set of high-confidence targets, it provides a cost-effective roadmap for downstream wet-lab experiments.

### 3.4. From Additive to Synergistic Modeling to Disentangle Polypharmacology

A fundamental characteristic of herbal medicine is polypharmacology, where therapeutic effects arise from non-linear, synergistic interactions among multiple targets rather than the simple additive sum of isolated effects. A critical question is whether NovelHTI implicitly assumes additive effects or truly captures these high-order dependencies.

We clarify that NovelHTI avoids the additive assumption ($Score_{A+B}=Score_{A}+Score_{B}$) through its GNN architecture. Unlike linear models that treat targets as independent variables, NovelHTI employs a non-linear message passing mechanism that models the conditional dependencies between proteins. In this framework, the embedding of a target is iteratively updated by aggregating information from its topological neighbors. Mathematically, this means the importance score assigned to Target *A* is dependent on the latent state of Target *B* within the same pathway motif.

Crucially, this architecture allows the model to prioritize functional modules over isolated hubs. If Target *A* and Target *B* function synergistically, their representations will be mutually reinforced during the graph convolution process. Consequently, the high-ranking targets output by NovelHTI constitute a computational synergistic module, which is a set of proteins that are co-activated by the symptom phenotype in a non-linear manner. This capability aligns with our observations in the Section 2.5 Case Study, where the model successfully identified multiple co-dependent regulators within the NF-κB pathway, rather than isolated hits. This distinguishes genuine mechanistic synergy from coincidental co-activation, providing a computational basis for understanding the holistic efficacy of TCM.

### 3.5. Limitations and Future Directions

While NovelHTI demonstrates robust predictive capability, we acknowledge several limitations inherent to computational systems pharmacology.

First, regarding mechanistic depth and causality, it is essential to distinguish between suggestive associations (computational predictions) and demonstrated mechanisms (biological facts). The herb-target links identified by our model represent statistical priorities derived from topological and semantic similarity. Although retrospective validation confirms that these predictions align with recent high-impact literature, they remain in silico inferences. Therefore, we explicitly state that NovelHTI provides a Target De-risking map. The definitive confirmation requires orthogonal in vivo assays, such as Western Blot or CRISPR-Cas9 knockout experiments.

Second, the model′s resolution is currently constrained by the static nature of the biological network. Interactions are inherently dynamic and tissue-specific. However, our current framework utilizes a consolidated graph that aggregates context-independent interactions. Regarding the quantification of this limitation, our Knowledge Erosion Analysis in Section 2.6 provides empirical evidence of robustness. The model maintains high performance (AUC >0.87) even when 30% of the structural links are randomly removed, suggesting that while dynamic temporal data is missing, the static topological signal is sufficiently dense to support reliable predictions. Future iterations will aim to integrate dynamic transcriptomic profiles to capture temporal variations.

Third, we address the challenge of Data Incompleteness in real-world scenarios. Newly discovered herbs often possess incomplete symptom profiles due to limited clinical documentation. While conventional rule-based models typically fail with such sparse inputs, NovelHTI mitigates this through its adaptive Bio-Contextual Fusion Gate. When specific symptom features are missing, the model′s attention mechanism dynamically re-distributes weights to the remaining available phenotypic features. This resilience is intrinsically supported by our “Cold-Start” experimental design in Section 2.1, where the model successfully inferred targets despite the complete absence of prior interaction history. Furthermore, the robustness analysis confirms that the architecture maintains high stability even under significant structural information loss, suggesting a generalized robustness to data sparsity typical of under-characterized natural products.

### 3.6. Generalizability Across Ethnopharmacological Systems

A pivotal question for the broader application of NovelHTI is its transferability beyond the specific context of TCM. While our current implementation is optimized for TCM-specific SymMap, the underlying Phenotype-to-Mechanism architecture possesses intrinsic generalizability.

To empirically validate this broad applicability and address potential dataset-specific biases, we performed a cross-database verification using the independent repository *HERB* [38]. Unlike SymMap, HERB aggregates data via distinct curation protocols. We randomly selected five representative herbs, *Scutellaria baicalensis*, *Salvia miltiorrhiza*, *Artemisia annua*, *Panax ginseng*, and *Ephedra sinica*, and cross-referenced their Top-20 predicted targets against HERB records. Remarkably, over 80% of our high-confidence predictions were corroborated by this external repository. This cross-database consistency confirms that NovelHTI captures universal pharmacological principles rather than overfitting to the specific annotation patterns of a single source.

Fundamentally, the biological interactome for PPIs and Pathways, serves as a universal invariant anchor. Regardless of whether a diagnosis originates from TCM, Ayurveda, or Western clinical phenotypes, the downstream molecular targets reside within the same human biological network. The challenge, therefore, lies not in the reasoning engine, but in the Semantic Alignment of diverse diagnostic vocabularies.

We propose that NovelHTI can be adapted to other systems through Ontology Alignment and Domain Adaptation. For Western medicine, the transition is relatively direct, as modern phenotypes defined by ICD-11 codes or HPO terms map atomically to biological functions. For other traditional systems like Ayurveda, distinct cultural concepts can be conceptually aligned via the *Unified Medical Language System (UMLS)*. However, we acknowledge that such alignment is non-trivial due to granularity mismatches. Traditional syndromes are holistic constructs that may not map one-to-one to atomic Western phenotypes, and the scarcity of structured symptom-herb databases in other ethnopharmacological systems remains a practical bottleneck.

Despite these data-level challenges, NovelHTI′s modular design allows the *Symptom Encoder* to be fine-tuned on new biological ontologies once such data becomes available, establishing a scalable roadmap for evolving from a TCM-specific tool into a cross-cultural platform for global drug discovery.

## 4. Materials and Methods

### 4.1. Study Design

To accelerate the **early-stage drug discovery** of natural products, this study establishes a streamlined in silico target deconvolution strategy, as outlined in Figure 1. The research framework is designed to mimic the logic of pharmaceutical development: (1) **Digitalization of Pharmacological Knowledge**, where scattered data on herbal phenotypes, molecular interactions, and signaling pathways are harmonized into a computable Pharmacological Space to resolve the complexity of herbal mixtures; (2) **Phenotype-Driven Target Deconvolution**, deploying the NovelHTI architecture to decode clinical indications (phenotypes) into molecular targets, acting as a virtual screening engine for cold-start herbs lacking experimental bioactivity data; (3) **Screening Reliability Assessment**, simulating diverse real-world drug discovery scenarios (e.g., data sparsity, imbalance) to validate the model′s utility as a robust prioritization tool for subsequent wet-lab validation.

### 4.2. Data Acquisition and Heterogeneous Graph Construction

#### 4.2.1. Pharmacological Data Curation and Standardization

To establish a biologically robust foundation for target deconvolution, we curated and standardized pharmacological data from multiple authoritative repositories. Interaction data linking traditional medicines to modern molecular targets were sourced from **SymMap** [33], a database specifically designed to bridge the gap between Traditional Chinese Medicine (TCM) phenotypes and modern biomedical entities.

To ensure the reliability of downstream predictions and mitigate potential annotation biases inherent in TCM databases, strict quality control (QC) protocols were implemented. This involved: (1) **Resolving Symptom Ambiguity:** All symptom descriptions were semantically aligned with the Unified Medical Language System (UMLS) and MeSH ontologies to resolve terminological discrepancies between TCM syndromes and modern phenotypes; (2) **Bias Mitigation:** We filtered out low-confidence entries (confidence score <0.8) and removed high-frequency generic symptoms (e.g., “general discomfort”) that lack discriminative pharmacological value; and (3) **Standardization:** Mapping all protein identifiers to the standardized UniProt nomenclature.

The final curated dataset comprises a multi-scale hierarchy of **698 Herbs, 2311 TCMSymptoms, 1143 MMSymptoms, 13,992 Diseases, and 7854 Targets**. These entities were mathematically formalized into adjacency matrices (e.g., $M_{\mathrm{HT}}$), representing the verified clinical associations between herbal interventions and phenotypic indications.

#### 4.2.2. Construction of the Pathway-Enhanced Systems Pharmacology Network

To capture the systemic mechanisms by which herbal components modulate biological networks, we constructed a heterogeneous graph G=(V,E). Unlike conventional models that treat proteins as isolated nodes, this graph integrates the **molecular interactome** with **functional pathway annotations** to simulate the complex physiological environment.

- **Node Set (V):** of **12,815 nodes** across two functional layers: (1) **7854 Target Nodes** (proteins) derived from SymMap; and (2) **4961 Pathway Nodes**, comprising 311 KEGG signaling pathways [39] and 4650 leaf-node Gene Ontology (GO) terms [40].
- **Edge Set (E):** To simulate biological information flow with high fidelity, we defined a total of **19 distinct edge types**.
  **1. Protein-Protein Interactions (PPIs):** Based on the classification by Menche et al. [36], we stratified PPIs into seven distinct categories to capture different mechanistic contexts:
  (1) **Binary:** Physical interactions derived from high-throughput screens (e.g., yeast two-hybrid).
  (2) **Complexes:** Stable protein complex assemblies.
  (3) **Kinase:** Directed kinase-substrate phosphorylation pairs.
  (4) **Literature:** High-confidence interactions curated from low-throughput experiments.
  (5) **Metabolic:** Enzyme-coupled metabolic reactions.
  (6) **Regulatory:** Transcription factors binding to regulatory elements.
  (7) **Signaling:** Directed signal transduction cascades.
  Among these, categories (3), (5), (6), and (7) are biologically **directional**. To capture feedback loops and bidirectional message passing in the GNN, we added 4 inverse edge types corresponding to these directional categories.
  **2. Target-Pathway Associations:** Proteins were explicitly linked to their functional contexts via four annotation types:
  (1) **KEGG** pathway membership;
  (2) GO **Biological Process**;
  (3) GO **Molecular Function**;
  (4) GO **Cellular Component**.
  To enable cross-scale feature propagation (from targets to pathways and back to targets), 4 inverse association types were included.
  Detailed definitions for all 19 edge types are provided in Table A1 in Appendix B. To ensure the reliability of the topological structure, we prioritized high-confidence interactions. For Protein-Protein Interactions (PPIs), raw data were sourced from the STRING database (v11.5). We applied a strict filtering criterion, retaining only interactions with a ′Combined Score ≥ 700′ (High Confidence). This threshold filters out low-throughput yeast two-hybrid noise, ensuring that the ′Binary′ and ′Literature′ edges in our graph represent experimentally verified physical contacts rather than putative associations. Edge weights were implicitly handled by the graph convolution mechanism, relying on the topological consistency of this high-confidence scaffold.

Additionally, self-loops were included for each target node to preserve intrinsic molecular properties.

### 4.3. The NovelHTI Framework

#### 4.3.1. Overview of the Virtual Screening Architecture

NovelHTI is engineered as an interpretable deep learning framework designed to perform in silico target deconvolution for medicinal herbs. By decoding clinical phenotypic signatures, the model identifies potential molecular targets without relying on pre-existing chemical structure data. As illustrated in Figure 1, the architecture mimics a systems pharmacology workflow, comprising four functionally distinct modules:

1. **Symptom-Driven Phenotypic Encoding**, which translates macroscopic clinical indications (TCMSymptoms) into computational feature embeddings;
2. **Multi-Scale Feature Integration**, which projects phenotypic features into the microscopic protein interaction space;
3. **Pathway-Enhanced Propagation**, which simulates biological signal transduction across the heterogeneous network to capture systemic regulatory mechanisms; and
4. **Interaction Scoring**, which quantitatively evaluates the binding probability between herbs and candidate targets.

To simulate the comparative nature of high-throughput screening, where the goal is to distinguish true active targets from non-interacting background proteins, we formulate the learning task as a pairwise ranking problem [41]. Specifically, the model is trained on triplet instances $\left(H,T_{p},T_{n}\right)$, where H represents the query herb, $T_{p}$ denotes a validated positive target (active), and $T_{n}$ denotes a negative decoy (inactive).

Mathematically, NovelHTI functions as a biological scoring function that maps these entities to interaction probabilities:

(1) $$s_{p},s_{n}=\mathrm{NovelHTI}\left(H,T_{p},T_{n}\right),$$

where $s_{p}\in \left[0,1\right]$ and $s_{n}\in \left[0,1\right]$ quantify the predicted interaction likelihood for the positive pair $\left(H,T_{p}\right)$ and the negative pair $\left(H,T_{n}\right)$, respectively. The optimization objective is to learn a manifold where the score of the experimentally validated target ($s_{p}$) is consistently higher than that of the decoy ($s_{n}$), thereby ensuring robust prioritization of potential therapeutic targets in real-world screening scenarios.

#### 4.3.2. Module 1: In Silico Digitalization of Herbal Phenotypes

A persistent challenge in natural product drug discovery is the chemical black box phenomenon, where the comprehensive molecular composition of a novel herb remains experimentally unresolved. To circumvent this dependency on prior chemical knowledge, we developed a phenotype-to-mechanism digitalization strategy that treats clinical indications (TCMSymptoms) as a high-dimensional therapeutic fingerprint. This approach effectively translates qualitative ethnomedical knowledge into quantitative pharmacological features, allowing for *de novo* analysis of unstudied herbs.

We first established a digitized efficacy profile for each herb by constructing a prior knowledge matrix $M_{\mathrm{HT}}\in {\left\{0,1\right\}}^{N_{h}\times N_{s}}$ derived from the SymMap database, where $N_{h}$ and $N_{s}$ represent the number of herbs and unique clinical symptoms, respectively. Within this matrix, an entry of $M_{\mathrm{HT}}\left(i,j\right)=1$ denotes a validated therapeutic association between herb *i* and symptom *j*. For any given query herb H, the model extracts a specific symptom vector $v_{pheno}$ from $M_{\mathrm{HT}}$. This vector serves as the herb′s macroscopic biological signature, encoding its therapeutic spectrum (e.g., specific inflammatory responses or metabolic regulations) without requiring molecular structural data.

To bridge the dimensional gap between macroscopic symptoms and microscopic molecular targets, the model projects this discrete phenotypic profile into a continuous latent feature space. Recognizing that clinical symptoms are external manifestations of underlying molecular perturbations, we employ a parameterized projection function $F_{proj}$ to capture the semantic relationships between distinct symptoms while simultaneously **filtering out sparsity-induced noise**:

(2) $$H_{\mathrm{enc}}=F_{proj}\left(v_{pheno}\right),$$

where $H_{\mathrm{enc}}$ represents the herb′s Digital Pharmacological Identity and $F_{proj}$ is implemented via a learnable dense layer (**projecting features to a latent dimension *d* aligned with the molecular target space**). This projection transforms the sparse, high-dimensional symptom vector into a dense representation that mathematically embodies the TCM principle of Treatment based on Pattern Differentiation, thereby providing a robust initialization for downstream target inference.

It is worth noting that while meta-paths define the semantic connectivity schema, our framework transcends simplistic linear cascades. Unlike sequential inference models, the heterogeneous graph attention mechanism aggregates information from all phenotypic and molecular neighbors simultaneously in a parallel manner. This architecture inherently captures biological pleiotropy, where a single symptom maps to diverse disease contexts and multiple targets and synergistic feedbacks. Consequently, NovelHTI learns complex, non-linear topological patterns rather than restricted single-chain deductions, effectively addressing the multifaceted nature of herbal interventions.

#### 4.3.3. Module 2: Multi-Scale Feature Integration via Biomedical Knowledge Cascades

While the previous module successfully digitizes the herb′s clinical symptoms, a significant semantic gap remains between these macroscopic phenotypes (e.g., Deficiency of Qi) and the microscopic molecular targets required for modern drug discovery. To bridge this disconnect, we constructed a translational knowledge bridge that propagates the herb′s phenotypic features through a hierarchical biological chain, effectively simulating the bedside-to-bench inference logic of systems pharmacology.

We model the association between a herb and its potential targets not as a direct jump, but as a causal cascade spanning four biological scales: *Traditional Symptom (TCM)* →*Modern Symptom (MM)*→*Disease*→*Molecular Target*. Formally, this inference process is implemented by propagating the initial herb embedding $H_{\mathrm{enc}}$ through a sequence of prior knowledge matrices. As the signal traverses from the phenotype space to the protein space, it is sequentially refined by the known associations between symptoms, diseases, and targets:

(3) $$\mathrm{Phenotype}\overset{M_{\mathrm{TM}}}{\to }\mathrm{Modern}\;\mathrm{Symptom}\overset{M_{\mathrm{MD}}}{\to }\mathrm{Disease}\overset{M_{\mathrm{DT}}}{\to }\mathrm{Target}\;\mathrm{Space},$$

where $M_{\mathrm{TM}}$, $M_{\mathrm{MD}}$, and $M_{\mathrm{DT}}$ represent the binary association matrices linking TCMSymptoms to MMSymptoms, MMSymptoms to Diseases, and Diseases to Targets, respectively.

This propagation mechanism is designed to be selective and path-dependent, ensuring that the herb′s latent features are transmitted only to targets that possess a mechanistic link to the herb′s specific indications. Consequently, rather than starting from a random distribution, the feature space for the 7854 candidate targets is biologically initialized: each target node receives a feature embedding $H_{\mathrm{target}}^{\left(0\right)}$ that explicitly reflects its potential relevance to the querying herb. By grounding the model′s initialization in this established biomedical knowledge cascade, we effectively narrow the search space and guide the subsequent graph neural network to focus on biologically plausible interactions. For detailed mathematical implementation and tensor operations, please refer to Appendix A.

To rigorously preclude information leakage, we enforced a structural separation between the general biomedical knowledge used for feature initialization and the specific pharmacological interactions serving as labels. The initialization cascade ($M_{\mathrm{TM}}\to M_{\mathrm{MM}}\to M_{\mathrm{DT}}$) is derived exclusively from herb-agnostic biomedical ontologies, rendering these pathological associations orthogonal to the specific Herb-Target bioactivity records ($M_{\mathrm{HT}}$). Combined with the strict cold-start protocol where test herb interactions were masked, this design compels the model to infer targets through phenotypic reasoning rather than memory retrieval, thereby ensuring the generalizability of the predicted mechanisms.

#### 4.3.4. Module 3: Simulation of Cross-Scale Signal Transduction

The core of NovelHTI is designed to mimic the biological reality that drug targets do not function in isolation, but rather within dynamic, interconnected signaling networks. Consequently, we devised a cross-scale signal transduction mechanism that propagates the initial pharmacological features (derived in Module 2) across the heterogeneous biological graph. As outlined in the algorithmic logic, this process executes an iterative update-and-refine cycle comprising four biologically coordinated stages:

**Stage 1: Simulating Local Protein Interactomes (Micro-Level Propagation)**

First, to capture the immediate micro-environment of potential targets, the model simulates signal exchange within the Protein-Protein Interaction (PPI) network. We employ a herb-specific attention mechanism, recognizing that different herbs modulate the same biological network with varying intensities. For a target node *v*, the regulatory intensity (attention weight) $\alpha_{r}^{\left(o,i\right)}$ is dynamically computed based on the herb′s specific phenotypic encoding:

(4) $$\alpha_{r}^{\left(o,i\right)}=\sigma \left(W_{\mathrm{attn}1}^{\left(o,i\right)}\cdot H_{\mathrm{enc}}\right).$$

This formulation ensures that the feature aggregation is context-aware: the model emphasizes PPI edges relevant to the herb′s therapeutic mechanism while suppressing irrelevant background noise. The aggregated signal $m_{r}^{\left(o,i\right)}\left(v\right)$ updates the target′s state, effectively modeling how local protein crosstalk influences drug efficacy.

**Stage 2: Integrating Systemic Pathway Context (Macro-Level Propagation)**

Simultaneously, to bridge the gap between individual proteins and holistic physiological functions, the model elevates information flow to the pathway level. Signals are propagated from target nodes to pathway nodes (e.g., KEGG/GO terms) and back. This dual message-passing step captures systemic feedback: a target is deemed more probable if it structurally belongs to a pathway highly activated by the herb′s symptom profile.

**Stage 3: Confidence-Guided Pharmacological Fusion**

A critical challenge in systems pharmacology is distinguishing meaningful biological signals from computational artifacts. To address this, we introduce a **Bio-Contextual Fusion Gate**. Instead of simply summing local (PPI) and global (Pathway) features, the model generates a learnable Confidence Map C to adaptively weigh the pathway information:

(5) $$F_{\mathrm{fused}}=C⊙\left(F_{\mathrm{target}}+F_{\mathrm{pathway}}^{\prime }\right),\;\mathrm{with}\;C=\sigma \left(F_{\mathrm{pathway}}\cdot W_{c}\right).$$

Here, C∈[0,1] acts as a biological gatekeeper, assigning high confidence to pathway features consistent with the target′s local topology. This ensures the refined representation $F_{\mathrm{fused}}$ is supported by both molecular evidence and systemic functional plausibility.

**Stage 4: Iterative Signal Refinement (Feedback Loop)**

Biological signal transduction is rarely a single-step process; it involves continuous feedback and amplification. To mimic this, the fused features $F_{\mathrm{fused}}$ from Stage 3 are not immediately output but are projected back to serve as the initialized state for the subsequent propagation round. Mathematically, the fused target representations are aligned with the pathway dimension via a linear projection and concatenated with the static pathway embeddings:

(6) $$F^{\left(o+1\right)}=\mathrm{UpdateState}\left(F_{\mathrm{fused}},P_{\mathrm{emb}}\right).$$

This re-injection mechanism allows the model to perform multi-hop reasoning, capturing deep semantic relationships (e.g., how a target indirectly influences a distant pathway) through iterative refinement, thereby enhancing the depth and accuracy of the final target prediction.

#### 4.3.5. Module 4: Interaction Scoring and Target Prioritization

Following feature propagation, the final module functions as a biological scoring engine. Its primary objective is to quantify the binding probability between the query herb and potential targets based on the refined pharmacological representations $F_{\mathrm{fused}}$ derived from the systems pharmacology network.

To quantify the therapeutic association for a specific herb-target pair, the model extracts the corresponding feature vector from the fused embedding space. Note that this embedding $e_{\mathrm{fused}}$ is not a static protein feature but a herb-conditioned representation, explicitly encoding the systemic crosstalk between the query herb and the candidate target. To capture the non-linear nature of biological interactions, we employ a projection function (MLP) to map this high-dimensional feature to a scalar interaction score:

(7) $$s=\mathrm{MLP}\left(e_{\mathrm{fused}}\right)=W_{2}\cdot \mathrm{ReLU}\left(W_{1}\cdot e_{\mathrm{fused}}+b_{1}\right)+b_{2}.$$

Crucially, rather than treating target identification as a binary classification task, NovelHTI generates a continuous probability ranking. By normalizing the raw score *s* via the sigmoid function σ(·), the model outputs a probability P∈[0,1], reflecting the likelihood of a therapeutic interaction. This design enables the framework to serve as a prioritization tool, providing experimentalists with a ranked list where top-scored proteins represent the most biologically plausible candidates for downstream wet-lab validation.

### 4.4. Optimization Strategy: Differentiating Actives from Decoys

To mimic the logic of high-throughput screening, where the goal is to distinguish true active targets from a vast background of non-interacting proteins, we formulated the model training as a discriminative ranking problem. However, since biological databases contain only positive samples, we employed a **Uniform Negative Sampling** strategy to generate decoys.

To mitigate potential sampling bias and ensure the decoys are representative of the inactive proteome, we implemented the following protocol:

1. **Unobserved Space Definition:** The negative pool was strictly defined as the complement of the verified interaction set and unobserved links, excluding any pairs with known validation.
2. **Dynamic Sampling:** We applied a Dynamic Sampling strategy during training. Instead of a fixed negative set, negative samples were re-drawn randomly from the unobserved space at the beginning of each training epoch. This prevents the model from overfitting to a specific set of easy negatives and forces it to learn robust features.
3. **Class Balance:** The ratio of positive to negative samples was maintained at 1:1 to enforce class balance, ensuring that the loss function is not dominated by the majority class.

Specifically, the model is optimized using a triplet-based strategy $\left(H,T_{\mathrm{active}},T_{\mathrm{decoy}}\right)$. Here, $T_{\mathrm{active}}$ represents a clinically validated target, while $T_{\mathrm{decoy}}$ represents a putative non-interacting background protein sampled via a negative sampling strategy. The optimization objective is to learn a pharmacological manifold where the predicted score of the active target is consistently higher than that of the decoy. We minimize the binary cross-entropy divergence to maximize this separation margin:

(8) $$L=-\frac{1}{N}\sum_{i=1}^{N}\left[\log\left(\sigma \left(s_{\mathrm{active}}^{\left(i\right)}\right)\right)+\log\left(1-\sigma \left(s_{\mathrm{decoy}}^{\left(i\right)}\right)\right)\right].$$

The loss function L forces the model to assign high probabilities to known therapeutic associations while suppressing the scores of background noise. To ensure robust convergence and prevent overfitting, a critical requirement for reliable pharmaceutical applications, the optimization is performed using the AdamW algorithm [42] with weight decay, which effectively regularizes the model parameters during the learning of complex biological patterns.

### 4.5. Stratified Benchmarking for Pharmacological Scenarios

To comprehensively evaluate the utility of NovelHTI in real-world drug discovery, we constructed seven stratified datasets (Datasets 00–06) derived from the SymMap database. A global statistical significance threshold of p≤0.05 was applied to ensure that all high-confidence associations possess sufficient statistical evidence. Recognizing that pharmacological data often exhibits complex distributions, we implemented two distinct sampling strategies to simulate different virtual screening scenarios.

First, to establish a controlled benchmarking environment, we employed a Standardized Baseline Evaluation strategy (Datasets 00, 01, 03, and 05). This method selects a fixed quota of highest-confidence interactions for each herb, creating a balanced dataset ideal for rigorously comparing algorithmic stability. Second, to address the long-tail distribution typical of natural product research, where well-studied herbs have abundant targets while others are data-scarce, we designed a Real-World Screening Simulation strategy (Datasets 02, 04, and 06). By adopting a min-for-each inclusion criterion, this approach maximizes data utilization and challenges the model to demonstrate robust performance across both data-rich and data-poor conditions. Detailed statistics for these pharmacological datasets are summarized in Table 8.

### 4.6. Baseline Comparisons and Rigorous Validation

Since herbal medicines are complex mixtures lacking a single defined chemical structure, conventional small-molecule embedding methods are inapplicable. To strictly validate the pharmacological predictive power of NovelHTI, we compared it against a comprehensive suite of advanced Knowledge Graph Embedding (KGE) models. These baselines were specifically selected for their capacity to handle the heterogeneous nature of biological networks (e.g., distinguishing semantic relations between Symptoms and Targets), a capability often lacking in standard homogeneous network algorithms. The comparative analysis includes Translational Distance Models (TransR [43], MuRE [44]), Semantic Matching Models (ComplEx [45], TuckER [46]), and Neural Interaction Models (ProjE [47], ConvE). Additionally, we included HTINet2 [23], a specialized framework for herb-target prediction, as the primary domain-specific competitor. All models were trained on the identical biological network topology to ensure a fair assessment.

To mitigate the impact of randomness and ensure the reproducibility required for pharmaceutical applications, we implemented a strict Multi-Seed Cross-Validation protocol. For each dataset, we performed independent evaluations across five random folds, with each fold repeated three times using distinct initialization seeds. A rigorous Early Stopping mechanism was enforced to prevent overfitting to specific data subsets, ensuring that the reported performance reflects the model′s true generalization capability to novel herbs.

Regarding evaluation metrics, while standard classification indicators (ROC-AUC, PR-AUC) provide a global view of model performance, they do not fully capture the practical constraints of drug discovery, where resources typically allow for validating only a few top-ranked candidates. Therefore, we prioritized Ranking-Based Metrics to assess the model′s enrichment factor. We focused on Hit Ratio (HR@N) and Normalized Discounted Cumulative Gain (NDCG@N), specifically reporting results for N∈{10,20} to reflect stringent screening criteria. High performance on these metrics indicates that the model can effectively narrow down the search space, providing experimentalists with a highly enriched list of biologically plausible targets for downstream validation.

To rigorously evaluate the proposed framework against state-of-the-art methodologies specifically in the computational pharmacology domain, we incorporated two additional categories of strong baselines. We implemented the HAN (Heterogeneous Graph Attention Network for DTI) architecture adapted for drug-target interaction tasks. By extending the inductive message-passing logic of GraphSAGE to heterogeneous networks, HAN serves as a robust benchmark to validate whether NovelHTI′s specific Symptom-Constraint logic outperforms generic graph attention mechanisms. Comparing against HAN allows us to verify whether NovelHTI′s Symptom-Constraint logic outperforms standard meta-path based aggregation in identifying therapeutic targets [48]. Following established benchmarks in drug discovery, we selected XGBoost (Feature-based DTI Baseline) as a strong non-GNN baseline. This gradient boosting model predicts targets solely based on symptom feature vectors without accessing the graph topology. Including this baseline allows us to explicitly quantify the performance gain attributed to topological signal propagation versus simple feature-based inference, validating the necessity of the PPI network structure [49].

## 5. Conclusions

In this study, we proposed NovelHTI, a systems pharmacology framework designed to bridge the gap between the clinical phenomenology of traditional medicine and the molecular mechanism of modern pharmacology. By constructing a translational link between macroscopic clinical symptoms and microscopic biological targets, this work introduces a Phenotype-to-Target strategy, facilitating the identification of potential therapeutic mechanisms for complex herbal formulations.

From the perspective of pharmaceutical R&D, comprehensive evaluations suggest that NovelHTI serves as a robust Virtual Gatekeeper for rational experimental design. By adopting a conservative prediction mode in data-sparse regimes, it helps distinguish authentic therapeutic associations from background noise. This strategy aims to prioritize candidates with high biological plausibility for wet-lab validation, thereby potentially reducing the attrition rate in early-stage discovery.

Mechanistically, the model captures the essence of herbal polypharmacology. By reconstructing the signal transduction architecture, integrating heterogeneous protein interactions and systemic pathway constraints, it successfully elucidates how holistic therapeutic effects emerge from the synergistic perturbation of molecular networks. Ultimately, NovelHTI provides a foundational platform for integrated ethnopharmacology. It accelerates the elucidation of complex Mechanisms of Action (MOA) for natural products and offers a strategic roadmap for designing next-generation multi-component combinatorial therapies.

## Figures and Tables

**Table 1:** Performance comparison of NovelHTI with other herb-target prediction methods. ROC-AUC, PR-AUC, Accuracy, and **F1-score** of NovelHTI and baseline models across three datasets are shown. All values are presented as percentages in the format of mean ± standard deviation.

Dataset	Metric	NovelHTI	HTINet2 + ERMLPE	HTINet2 + PairRE	HTINet2 + TuckER	HTINet2 + MuRE
	ROC-AUC	85.36 ± 1.64	51.47 ± 2.96	50.80 ± 3.72	50.73 ± 3.45	50.37 ± 4.05
**Dataset 02**	PR-AUC	87.18 ± 1.19	63.13 ± 2.68	62.67 ± 2.74	62.34 ± 2.98	61.95 ± 2.94
	Accuracy	79.46 ± 1.38	50.39 ± 1.66	50.35 ± 2.36	50.51 ± 2.74	50.59 ± 2.22
	F1-score	**77.96 ± 1.50**	50.15 ± 1.80	50.10 ± 2.40	50.25 ± 2.80	50.30 ± 2.30
	ROC-AUC	94.86 ± 1.01	50.18 ± 3.09	46.39 ± 6.08	51.79 ± 6.28	49.61 ± 5.16
**Dataset 04**	PR-AUC	95.56 ± 0.64	66.56 ± 2.25	63.65 ± 4.71	67.35 ± 4.33	65.85 ± 3.85
	Accuracy	91.53 ± 0.93	49.86 ± 1.25	48.60 ± 2.04	50.26 ± 2.52	49.63 ± 2.15
	F1-score	**91.36 ± 1.05**	49.50 ± 1.30	48.20 ± 2.10	50.10 ± 2.60	49.40 ± 2.20
	ROC-AUC	95.04 ± 1.71	50.02 ± 4.54	48.33 ± 5.53	50.71 ± 6.00	51.10 ± 4.64
**Dataset 06**	PR-AUC	95.69 ± 1.28	66.17 ± 3.43	65.03 ± 4.17	66.47 ± 4.48	66.90 ± 3.59
	Accuracy	91.51 ± 2.03	49.72 ± 1.73	49.48 ± 2.25	50.50 ± 2.28	50.10 ± 2.11
	F1-score	**91.86 ± 1.95**	49.40 ± 1.80	49.10 ± 2.30	50.20 ± 2.40	49.90 ± 2.15
**Dataset**	**HTINet2 + TransR**	**HTINet2 + ComplEx**	**HTINet2 + ProjE**	**HTINet2 + CrossE**	**HAN**	**XGBoost**
	49.95 ± 3.77	49.88 ± 5.27	49.49 ± 5.20	49.30 ± 5.10	78.42 ± 2.10	68.95 ± 1.20
**Dataset 02**	61.83 ± 2.98	61.58 ± 4.47	61.62 ± 3.68	61.65 ± 4.08	75.30 ± 1.88	65.40 ± 1.55
	49.61 ± 2.34	49.81 ± 2.91	49.75 ± 3.21	49.49 ± 3.16	71.20 ± 1.92	62.15 ± 1.40
	49.50 ± 2.50	49.60 ± 3.00	49.55 ± 3.30	49.20 ± 3.20	70.85 ± 1.95	61.90 ± 1.45
	49.53 ± 6.36	53.31 ± 5.74	49.40 ± 5.36	48.80 ± 5.69	86.55 ± 1.42	74.20 ± 0.95
**Dataset 04**	65.57 ± 4.90	68.52 ± 3.85	65.61 ± 3.82	64.85 ± 3.94	84.10 ± 1.10	72.80 ± 1.12
	50.27 ± 2.29	51.13 ± 1.96	49.29 ± 1.63	50.49 ± 1.46	81.25 ± 1.50	68.50 ± 1.10
	49.80 ± 2.40	50.90 ± 2.10	49.10 ± 1.70	50.20 ± 1.50	80.95 ± 1.55	68.20 ± 1.15
	48.16 ± 4.79	51.72 ± 5.37	48.19 ± 5.01	50.54 ± 4.43	87.10 ± 1.65	75.15 ± 1.05
**Dataset 06**	64.33 ± 4.43	67.45 ± 4.03	64.83 ± 3.66	66.50 ± 3.17	84.50 ± 1.35	73.20 ± 1.25
	50.25 ± 1.93	50.30 ± 1.97	49.34 ± 2.16	49.95 ± 1.62	81.80 ± 1.85	69.10 ± 1.30
	49.90 ± 2.00	50.10 ± 2.10	49.10 ± 2.20	49.80 ± 1.70	81.55 ± 1.90	68.85 ± 1.35

*Note:* Statistical significance was evaluated using the paired Wilcoxon signed-rank test. The performance improvement of NovelHTI over the best baseline (HTINet2 variants) is statistically significant with p<0.01 across all datasets.

**Table 2:** Performance of NovelHTI across 6 datasets. All values are presented as percentages in the format of mean ± standard deviation.

Dataset	ROC-AUC	PR-AUC	Accuracy	Precision	Recall	F1-Score
Dataset 05	97.70±0.72	97.76±0.72	94.80±1.17	94.69±1.66	94.97±2.05	94.83±1.55
Dataset 03	95.40±1.33	95.99±0.93	91.70±1.39	92.98±2.22	90.31±3.21	91.62±2.10
Dataset 06	95.04±1.71	95.69±1.28	91.51±2.03	93.17±1.87	90.60±3.36	91.86±1.95
Dataset 04	94.86±1.01	95.56±0.64	91.53±0.93	93.35±0.93	89.46±2.17	91.36±1.05
Dataset 01	88.71±2.04	88.98±2.58	83.53±3.32	85.70±3.05	80.74±8.00	83.10±4.50
Dataset 02	85.36±1.64	87.18±1.19	79.46±1.38	84.25±1.39	72.55±3.94	77.96±1.50

*Note:* Statistical significance was evaluated using the paired Wilcoxon signed-rank test. The performance improvement of NovelHTI over the best baseline (HTINet2 variants) is statistically significant with p<0.01 across all datasets.

**Table 3:** Ablation study for NovelHTI on dataset 05. All values are presented as percentages in the format of mean ± standard deviation.

Model	ROC-AUC	PR-AUC	Accuracy	Precision	Recall
NovelHTI	97.70 ± 0.72	97.76 ± 0.72	94.80 ± 1.17	94.69 ± 1.66	94.97 ± 2.05
NO_multi_ppis	93.94 ± 1.98	93.64 ± 1.71	88.09 ± 2.73	88.78 ± 2.47	87.23 ± 4.35
NO_hGCN	93.85 ± 5.22	94.12 ± 4.36	88.99 ± 5.26	89.75 ± 8.15	89.31 ± 4.42
NO_pathways	90.25 ± 12.48	92.96 ± 6.61	85.09 ± 11.88	88.23 ± 10.46	84.30 ± 24.35

**Table 4:** Ablation study for NovelHTI on dataset 03. All values are presented as percentages in the format of mean ± standard deviation.

Model	ROC-AUC	PR-AUC	Accuracy	Precision	Recall
NovelHTI	95.40 ± 1.33	95.99 ± 0.93	91.70 ± 1.39	92.98 ± 2.22	90.31 ± 3.21
NO_hGCN	94.38 ± 1.53	94.99 ± 1.38	89.89 ± 1.84	91.92 ± 3.61	87.78 ± 4.62
NO_pathways	94.10 ± 2.23	94.69 ± 1.88	89.92 ± 3.09	92.66 ± 3.84	86.99 ± 6.68
NO_multi_ppis	85.78 ± 2.77	84.32 ± 3.20	78.68 ± 2.62	80.17 ± 3.12	76.43 ± 5.54

**Table 5:** Ablation study for NovelHTI on dataset 01. All values are presented as percentages in the format of mean ± standard deviation.

Model	ROC-AUC	PR-AUC	Accuracy	Precision	Recall
NovelHTI	88.71 ± 2.04	88.98 ± 2.58	83.53 ± 3.32	85.70 ± 3.05	80.74 ± 8.00
NO_pathways	87.71 ± 5.52	88.58 ± 5.93	82.75 ± 6.62	83.77 ± 9.14	83.32 ± 6.08
NO_hGCN	84.38 ± 9.88	85.27 ± 4.81	79.11 ± 8.54	75.74 ± 21.32	76.21 ± 21.74
NO_multi_ppis	67.46 ± 6.84	66.34 ± 6.77	62.82 ± 5.93	64.32 ± 6.64	58.37 ± 13.97

**Table 6:** **External validation of NovelHTI predictions for** ***Scutellaria baicalensis*** **using post-training literature (2021–2024).** The table lists high-confidence targets predicted by NovelHTI and their corresponding experimental validations published in recent independent studies. Note that the validation years (2021–2024) post-date the training data, ensuring a rigorous temporal blind test.

Predicted Rank	Target	Validation Year	Experimental Method	Observed Biological Effect	Ref.
Top 0.1%	NLRP3	**2024**	Western Blot, Immunofluorescence	Inhibition of NLRP3 inflammasome activation and pyroptosis	[30]
Top 0.1%	PTGS2	**2022**	qPCR, Western Blot	Downregulation of Ptgs2 (COX-2) mRNA and ferroptosis markers	[29]
Top 0.1%	RELA	**2021**	Western Blot, ELISA	Suppression of NF-κB p65 pathway activation and inflammation	[28]
Top 0.5%	MAPK1	**2022**	Western Blot	Suppression of ERK1/2 (MAPK) phosphorylation	[31]
Top 0.5%	TNF	**2023**	ELISA, ROS assay	Diminished production of TNF-α and neutrophil inflammation	[32]

**Table 7:** Dual-Validation of NovelHTI: Retrospective prioritization of Canonical Consensus (Robustness) and Emerging Mechanisms (Predictiveness) against post-2020 literature.

Herb	Validation Type	Target (Predicted Rank)	Status in 2019	Year	Ref.
*S. miltiorrhiza*	**Robustness** (Anchor)	**NLRP3 (8)**	Latent/Noisy	2023	[35]
*S. miltiorrhiza*	Robustness	GSDMD (14)	Latent	2023	[35]
*A. annua*	**Predictiveness** (Blind)	**GPX4 (4)**	**Novel**	2022	[34]

**Table 8:** Statistical Stratification of Benchmarking Datasets. The table details the confidence thresholds and sampling strategies used to construct datasets representing varying degrees of pharmacological evidence and data sparsity.

Dataset	Min Conf.	Max Conf.	Sampling Strategy	Herbs	Min Triplets	Max Triplets	Total Triplets
Dataset 00	0.180	0.500	Balanced (Fixed-20)	354	20	20	7080
Dataset 01	0.200	0.250	Balanced (Fixed-2)	576	2	2	1152
Dataset 02	0.200	0.250	Imbalanced (Maximal)	576	2	652	48,534
Dataset 03	0.175	0.550	Balanced (Fixed-5)	447	5	5	2235
Dataset 04	0.175	0.550	Imbalanced (Maximal)	447	5	249	16,423
Dataset 05	0.175	0.550	Balanced (Fixed-20)	310	20	20	6200
Dataset 06	0.175	0.550	Imbalanced (Maximal)	310	20	249	14,904

## Data Availability

The datasets generated and analyzed during the current study are available in the **Zenodo** repository (DOI: 10.5281/zenodo.15555399). To ensure full transparency and reproducibility, the comprehensive source code—including the exact model architecture, hyperparameter configurations (e.g., specific layer dimensions, learning rates), and random seed settings—is openly available on **GitHub** at https://github.com/CheungYuyam/NovelHTI.git (accessed on 18 February 2026).

## Supplementary Materials

The following supporting information can be downloaded at: https://www.mdpi.com/article/10.3390/ph19030413/s1, Section S1: Hyperparameters search for NovelHTI. Figure S1: Hypermeter search for layer_*i*_ and layer_*o*_. Table S1: Detailed metrics of hypermeter search for dim1. Table S2: Detailed metrics of hypermeter search for dim2. Table S3: Detailed metrics of hypermeter search for layer_*i*_. Table S4: Detailed metrics of hypermeter search for layer_*o*_. Table S5: Performance comparison of NovelHTI with baseline models on dataset 02. Table S6: Performance comparison of NovelHTI with baseline models on dataset 04. Table S7: Performance comparison of NovelHTI with baseline models on dataset 06.

## Appendix A. Detailed Mathematical Implementation of the Feature Integration Module

This note provides the rigorous mathematical formulation and tensor operations corresponding to **Module 2** (Multi-Scale Feature Integration) described in the main text. While the main text outlines the biological logic, the specific computational implementation involves high-dimensional tensor transformations and sparse matrix operations.

### Appendix A.1. Tensor Initialization via Symptom Expansion

Let $H_{\mathrm{enc}}\in R^{B\times d}$ denote the batch of herb embeddings generated by Module 1, where *B* is the batch size and *d* is the latent feature dimension. The prior knowledge matrix linking herbs to TCMSymptoms is denoted as $M_{\mathrm{HT}}\in {\left\{0,1\right\}}^{N_{h}\times N_{s}}$, where $N_{h}=698$ and $N_{s}$ = 2311.

To initiate the propagation, we first perform a selective tensor expansion operation (mathematically equivalent to the zero-padding described in previous literature). For a specific herb *h* in the batch with index idx, its expanded symptom tensor $T_{\mathrm{symptom}}^{\left(h\right)}\in R^{N_{s}\times d}$ is constructed as follows:

(A1) $$T_{\mathrm{symptom}}^{\left(h\right)}\left[j,:\right]=\left\{\begin{array}{cc} H_{\mathrm{enc}}\left[idx,:\right] & \mathrm{if}\;M_{\mathrm{HT}}\left(idx,j\right)=1\\ 0 & \mathrm{if}\;M_{\mathrm{HT}}\left(idx,j\right)=0 \end{array}\right.,\;\forall j\in \left\{1,\ldots ,N_{s}\right\}$$

This operation broadcasts the herb′s latent embedding only to the dimensions corresponding to its valid clinical symptoms, while zero-padding all irrelevant dimensions. Applying this to the entire batch yields the initial 3D symptom feature tensor $X_{0}\in R^{B\times N_{s}\times d}$.

### Appendix A.2. Sequential Matrix Propagation

The feature propagation through the biological chain (*TCMSymptom* →*MMSymptom*→*Disease*→ *Target*) is implemented via sequential matrix multiplications along the second dimension of the tensor.

We define the following bipartite association matrices based on SymMap data:

- $M_{\mathrm{TM}}\in {\left\{0,1\right\}}^{N_{s}\times N_{mm}}$ (2311 × 1143)
- $M_{\mathrm{MD}}\in {\left\{0,1\right\}}^{N_{mm}\times N_{dis}}$ (1143 × 13,992)
- $M_{\mathrm{DT}}\in {\left\{0,1\right\}}^{N_{dis}\times N_{tgt}}$ (13,992 × 7854)

The computational flow proceeds as follows:

1. **Mapping to MMSymptom Space:**
  (A2) $$X_{1}=X_{0}\times M_{\mathrm{TM}},\;\mathrm{where}\;X_{1}\in R^{B\times 1,143\times d}$$
2. **Mapping to Disease Space:**
  (A3) $$X_{2}=X_{1}\times M_{\mathrm{MD}},\;\mathrm{where}\;X_{2}\in R^{B\times 13,992\times d}$$
3. **Mapping to Target Space (Final Initialization):**
  (A4) $$X_{\mathrm{target}}=X_{2}\times M_{\mathrm{DT}},\;\mathrm{where}\;X_{\mathrm{target}}\in R^{B\times 7,854\times d}$$

The resulting tensor $X_{\mathrm{target}}$ serves as the initialized feature matrix for the target nodes in the subsequent Graph Neural Network module. This ensures that each target node′s initial feature vector is not random, but a rigorous aggregation of the pharmacological features derived from the herb′s phenotype-disease lineage.

## Appendix B. Detailed Definition of Heterogeneous Edge Types

To capture the complex Mechanism of Action (MoA) of herbal compounds, NovelHTI constructs a heterogeneous graph comprising 19 distinct edge types. Unlike homogeneous networks that treat all interactions equally, we stratified biological relationships based on the classification by Menche et al. [36] to preserve the mechanistic fidelity of the interactome.

**Table A1:** **Detailed breakdown of the 19 edge types used in NovelHTI.** The graph includes mechanism-stratified PPIs and Target-Pathway associations.

Category	Relation	Dir.	Data Source & Criteria	Pharmacological Relevance (MoA)
***A. Protein-Protein Interactions (PPIs)***				
**Physical**	(1) Binary	-	BioGRID; Score ≥ 0.7	**Direct Binding:** Identifies steric hindrance targets.
	(2) Complex	-	CORUM; PDB	**Assemblies:** Targets stable protein complexes.
**Regul.**	(3) Kinase	→	PhosphoSitePlus	**Signal Transduction:** Kinase inhibitor discovery.
	(4) Reg.	→	TRANSFAC	**Gene Modulation:** Transcription factor binding.
	(5) Signal	→	Signor	**Cascades:** Downstream effector pathways.
	(6) Metabolic	→	KEGG Metabolic	**Enzyme Kinetics:** Models ADME/metabolic flux.
**Curated**	(7) Lit.	-	Medline Mining	**Gold Standard:** Validated interactions.
**Inverse**	(8)–(11)	→	Derived from (3)–(6)	**Feedback:** Captures homeostatic regulation.
***B. Target-Pathway Associations***				
**Func.**	(12) KEGG	→	KEGG Database	**Systemic Context:** Links targets to disease axes.
	(13) GO:BP	→	GO Bio. Process	**Physiology:** Macroscopic biological outcomes.
	(14) GO:MF	→	GO Mol. Function	**Biochemistry:** Specific molecular activities.
	(15) GO:CC	→	GO Cell Comp.	**Accessibility:** Subcellular localization.
**Inverse**	(16)–(19)	→	Derived from (12)–(15)	**Inference:** Enables pathway-to-target reasoning.

*Note:* Dir. indicates Direction (→: Directed; -: Undirected). Regul. = Regulatory; Func. = Functional. Data sources were filtered using a combined confidence score ≥ 700 to ensure topological reliability.

## References

[1] Lv Q., Chen G., He H., Yang Z., Zhao L., Zhang K., Chen C.Y.C. (2023). TCMBank-the largest TCM database provides deep learning-based Chinese-Western medicine exclusion prediction. Signal Transduct. Target. Ther..

[2] Li X.L., Zhang J.Q., Shen X.J., Zhang Y., Guo D.A. (2025). Overview and limitations of database in global traditional medicines: A narrative review. Acta Pharmacol. Sin..

[3] Li C., Jia W.w., Yang J.l., Cheng C., Olaleye O.E. (2022). Multi-compound and drug-combination pharmacokinetic research on Chinese herbal medicines. Acta Pharmacol. Sin..

[4] Gan X., Shu Z., Wang X., Yan D., Li J., Ofaim S., Albert R., Li X., Liu B., Zhou X. (2023). Network medicine framework reveals generic herb-symptom effectiveness of traditional Chinese medicine. Sci. Adv..

[5] Xu Z. (2011). Modernization: One step at a time. Nature.

[6] Jiang Y., Wei S., Ge H., Zhang Y., Wang H., Wen X., Guo C., Wang S., Chen Z., Li P. (2025). Advances in the Identification Methods of Food-Medicine Homologous Herbal Materials. Foods.

[7] Huang X., Kong L., Li X., Chen X., Guo M., Zou H. (2004). Strategy for analysis and screening of bioactive compounds in traditional Chinese medicines. J. Chromatogr. B.

[8] Chen B., Liu S., Xia H., Li X., Zhang Y. (2025). Computer-Aided Drug Design in Research on Chinese Materia Medica: Methods, Applications, Advantages, and Challenges. Pharmaceutics.

[9] Dandibhotla S., Samudrala M., Kaneriya A., Dakshanamurthy S. (2025). GNNSeq: A Sequence-Based Graph Neural Network for Predicting Protein–Ligand Binding Affinity. Pharmaceuticals.

[10] Wang J., Xu S., Mei Y., Cai S., Gu Y., Sun M., Liang Z., Xiao Y., Zhang M., Yang S. (2021). A high-quality genome assembly of Morinda officinalis, a famous native southern herb in the Lingnan region of southern China. Hortic. Res..

[11] Nguyen T.M., Nguyen T., Tran T. (2022). Mitigating cold-start problems in drug-target affinity prediction with interaction knowledge transferring. Briefings Bioinform..

[12] Liu Z., Wang X.N., Yu H., Shi J.Y., Dong W.M. (2022). Predict multi-type drug–drug interactions in cold start scenario. BMC Bioinform..

[13] Ye Q., Hsieh C.Y., Yang Z., Kang Y., Chen J., Cao D., He S., Hou T. (2021). A unified drug–target interaction prediction framework based on knowledge graph and recommendation system. Nat. Commun..

[14] Li M., Wang Y., Ni Y., Xiong H., Mei Z., Zhang W. (2025). Revealing Herb-Symptom Associations and Mechanisms of Action in Protein Networks Using Subgraph Matching Learning. IEEE J. Biomed. Health Inform..

[15] Chandak P., Huang K., Zitnik M. (2023). Building a knowledge graph to enable precision medicine. Sci. Data.

[16] Rao A., Vg S., Joseph T., Kotte S., Sivadasan N., Srinivasan R. (2018). Phenotype-driven gene prioritization for rare diseases using graph convolution on heterogeneous networks. BMC Med. Genom..

[17] Gaudelet T., Day B., Jamasb A.R., Soman J., Reau C., Liu G., Hayter J.B.R., Vickers R., Roberts C., Tang J. (2021). Utilizing graph machine learning within drug discovery and development. Briefings Bioinform..

[18] Luo H., Yin W., Wang J., Zhang G., Liang W., Luo J., Yan C. (2024). Drug-drug interactions prediction based on deep learning and knowledge graph: A review. Iscience.

[19] Yu Y., Huang K., Zhang C., Glass L.M., Sun J., Xiao C. (2021). SumGNN: Multi-typed drug interaction prediction via efficient knowledge graph summarization. Bioinformatics.

[20] Zhang Y., Yao Q., Yue L., Wu X., Zhang Z., Lin Z., Zheng Y. (2023). Emerging drug interaction prediction enabled by a flow-based graph neural network with biomedical network. Nat. Comput. Sci..

[21] Zhu Y., Ren L., Sun R., Wang J., Yu G. (2024). Herb-Target Interaction Prediction by Multi-instance Learning. IEEE Trans. Artif. Intell..

[22] Zhang L., Li M., Shi X., Wang L. (2024). MT-HTI: A novel approach based on metapath2Vec and transformer for herb-target interaction prediction. Proceedings of the Fourth International Conference on Biomedicine and Bioinformatics Engineering (ICBBE 2024).

[23] Duan P., Yang K., Su X., Fan S., Dong X., Zhang F., Li X., Xing X., Zhu Q., Yu J. (2024). HTINet2: Herb–target prediction via knowledge graph embedding and residual-like graph neural network. Briefings Bioinform..

[24] Wang N., Li P., Hu X., Yang K., Peng Y., Zhu Q., Zhang R., Gao Z., Xu H., Liu B. (2019). Herb target prediction based on representation learning of symptom related heterogeneous network. Comput. Struct. Biotechnol. J..

[25] Song Z., Chen G., Chen C.Y.C. (2024). AI empowering traditional Chinese medicine?. Chem. Sci..

[26] Wu S., Sun F., Zhang W., Xie X., Cui B. (2022). Graph neural networks in recommender systems: A survey. ACM Comput. Surv..

[27] Zhang P., Zhang D., Zhou W., Wang L., Wang B., Zhang T., Li S. (2024). Network pharmacology: Towards the artificial intelligence-based precision traditional Chinese medicine. Briefings Bioinform..

[28] Fu Y., Xu B., Huang S., Luo X., Deng X., Luo S., Liu C., Wang Q., Chen J., Zhou L. (2021). Baicalin prevents LPS-induced activation of TLR4/NF-*κ*B p65 pathway and inflammation in mice via inhibiting the expression of CD14. Acta Pharmacol. Sin..

[29] Wang I.C., Lin J.H., Lee W.S., Liu C.H., Lin T.Y., Yang K.T. (2023). Baicalein and luteolin inhibit ischemia/reperfusion-induced ferroptosis in rat cardiomyocytes. Int. J. Cardiol..

[30] Liu Z., Dang B., Li Z., Wang X., Liu Y., Wu F., Cao X., Wang C., Lin C. (2024). Baicalin attenuates acute skin damage induced by ultraviolet B via inhibiting pyroptosis. J. Photochem. Photobiol. B.

[31] Kim K.A., Jung J.H., Choi Y.S., Kim S.T. (2022). Wogonin inhibits tight junction disruption via suppression of inflammatory response and phosphorylation of AKT/NF-*κ*B and ERK1/2 in rhinovirus-infected human nasal epithelial cells. Inflamm. Res..

[32] Li L., Dong J., Ye H., Jiang M., Yang H., Liang L., Ning L., Wu Y. (2023). Baicalin promotes antiviral IFNs production and alleviates type I IFN-induced neutrophil inflammation. J. Nat. Med..

[33] Wu Y., Zhang F., Yang K., Fang S., Bu D., Li H., Sun L., Hu H., Gao K., Wang W. (2019). SymMap: An integrative database of traditional Chinese medicine enhanced by symptom mapping. Nucleic Acids Res..

[34] Song Q., Peng S., Che F., Zhu X. (2022). Artesunate induces ferroptosis via modulation of p38 and ERK signaling pathway in glioblastoma cells. J. Pharmacol. Sci..

[35] Gao S., Liu Z., Li H., Little P., Liu P., Xu S. (2023). Cardiovascular actions and therapeutic potential of tanshinone IIA. Signal Transduct. Target. Ther..

[36] Menche J., Sharma A., Kitsak M., Ghiassian S.D., Vidal M., Loscalzo J., Barabási A.L. (2015). Uncovering disease-disease relationships through the incomplete interactome. Science.

[37] Barabási A.L., Gulbahce N., Loscalzo J. (2011). Network medicine: A network-based approach to human disease. Nat. Rev. Genet..

[38] Fang S., Dong L., Liu L., Guo J., Zhao L., Zhang J., Bu D., Liu X., Huo P., Cao W. (2021). HERB: A high-throughput experiment-and reference-guided database of traditional Chinese medicine. Nucleic Acids Res..

[39] Kanehisa M., Furumichi M., Sato Y., Matsuura Y., Ishiguro-Watanabe M. (2025). KEGG: Biological systems database as a model of the real world. Nucleic Acids Res..

[40] Ashburner M., Ball C.A., Blake J.A., Botstein D., Butler H., Cherry J.M., Davis A.P., Dolinski K., Dwight S.S., Eppig J.T. (2000). Gene ontology: Tool for the unification of biology. Nat. Genet..

[41] Zitnik M., Agrawal M., Leskovec J. (2018). Modeling polypharmacy side effects with graph convolutional networks. Bioinformatics.

[42] Loshchilov I., Hutter F. (2017). Decoupled weight decay regularization. arXiv.

[43] Lin Y., Liu Z., Sun M., Liu Y., Zhu X. (2015). Learning entity and relation embeddings for knowledge graph completion. Proceedings of the AAAI Conference on Artificial Intelligence.

[44] Balazevic I., Allen C., Hospedales T. (2019). Multi-relational poincaré graph embeddings. Adv. Neural Inf. Process. Syst..

[45] Trouillon T., Welbl J., Riedel S., Gaussier É., Bouchard G. (2016). Complex embeddings for simple link prediction. Proceedings of the International Conference on Machine Learning.

[46] Tucker L.R. (1966). Some mathematical notes on three-mode factor analysis. Psychometrika.

[47] Shi B., Weninger T. (2017). Proje: Embedding projection for knowledge graph completion. Proceedings of the AAAI Conference on Artificial Intelligence.

[48] Yu L., Qiu W., Lin W., Cheng X., Xiao X., Dai J. (2022). HGDTI: Predicting drug–target interaction by using information aggregation based on heterogeneous graph neural network. BMC Bioinform..

[49] Thafar M.A., Olayan R.S., Ashoor H., Albaradei S., Bajic V.B., Gao X., Gojobori T., Essack M. (2020). DTiGEMS+: Drug–target interaction prediction using graph embedding, graph mining, and similarity-based techniques. J. Cheminformatics.

