# Supplementary material for: NovelHTI: An Interpretable Pathway-Enhanced Framework for De Novo Target Prediction of Medicinal Herbs via Cross-Scale Heterogeneous Information Fusion
Source: Pharmaceuticals (Basel). 2026 Mar 3;19(3):413. doi: 10.3390/ph19030413 (PMC13028873; doi:10.3390/ph19030413)
Supplement: Supplementary file 1 [file pharmaceuticals-19-00413-s001.zip › pharmaceuticals-4128041-supplementary.pdf]

Pathway enhanced cross-scale interpretable model for "novel herb"-target interaction prediction based on heterogeneous graph convolutional neural networks

Supplementary Information

Contents

Supplementary Sections **2**

Supplementary Section S1 Hyperparameters search for NovelHTI 2

Supplementary Figures **3**

Figure S1 Hypermeter search for layer_i and layer_o. 3

Supplementary Tables **4**

Table S1 Detailed metrics of hypermeter search for dim1. 4

Table S2 Detailed metrics of hypermeter search for dim2. 4

Table S3 Detailed metrics of hypermeter search for layer_i. 4

Table S4 Detailed metrics of hypermeter search for layer_o. 4

Table S5 Performance comparison of NovelHTI with baseline models on dataset 02. 5

Table S6 Performance comparison of NovelHTI with baseline models on dataset 04. 5

Table S7 Performance comparison of NovelHTI with baseline models on dataset 06. 5

Supplementary Sections

Supplementary Section S1 Hyperparameters search for NovelHTI

Figure2 and Supplementary Table S1 illustrates the hyperparameter search results for NovelHTI. The search initially focused on dim1, with dim2 fixed at 8, and both layer_i and layer_o set to 3. As shown in Figure2A, the ROC-AUC exhibited a clear upward trend as dim1 increased from 2 to 16, stabilizing at higher values. Specifically, a dim1 value of 2 yielded an average ROC-AUC of 83.07 ± 11.64(%), while increasing it to 4 improved performance to 93.18 ± 1.65(%). Further increments to 8 and 16 achieved ROC-AUC values of 93.15 ± 5.70(%) and 97.01 ± 0.76(%), respectively. Notably, dim1 = 16 demonstrated both the highest mean performance (97.01%) and the lowest standard deviation (±0.76%), indicating superior robustness. Based on these observations, dim1 = 16 was selected as the optimal configuration for subsequent experiments.

The hyperparameter search then proceeded to dim2, with dim1 fixed at the previously determined 16, and layer_i and layer_o remaining at 3. As shown in Figure2B and Supplementary Table S2, dim2 = 4 achieved a mean ROC-AUC of 96.73 ± 0.97(%), while doubling the parameter to 8 resulted in a slight decline to 96.48 ± 0.52(%). Further escalation to 16 yielded marginal gains (96.76 ± 1.10(%)). Notably, dim2 = 8 demonstrated the lowest standard deviation (±0.52%), indicating superior stability despite a marginally lower mean ROC-AUC compared to dim2 = 4 (96.73% vs. 96.48%). This trade-off highlights the balance between peak performance and robustness in real-world applications. Given the emphasis on generalizability and reduced variance for practical deployment, dim2 = 8 was selected as the optimal configuration.

Finally, with dim1 and dim2 fixed at 16 and 8, respectively, the search focused on optimizing layer_i and layer_o. Initial experiments exploring values from 1 to 3 for both parameters (9 configurations in total) revealed a pronounced upward trend in performance as their depths increased (Supplementary FigureS1). To further refine the selection, two subsequent searches were conducted: (1) fixing layer_i at 3 while varying layer_o from 1 to 4 (Figure2C and Supplementary Table S3), and (2) fixing layer_o at 3 while varying layer_i from 1 to 4 (Figure2D and and Supplementary Table S4). Increasing layer_i from 1 to 4 improved the mean ROC-AUC from 93.78 ± 2.13(%) to 96.87 ± 0.79(%). Similarly, escalating layer_o from 1 to 4 boosted performance from 91.38 ± 2.54(%) to 97.40 ± 0.73(%). Notably, layer_o = 4 achieved the highest mean ROC-AUC (97.40%) with the lowest variance (±0.73%), significantly outperforming other configurations. However, simultaneous use of layer_i = 4 and layer_o = 4 was prohibited by GPU memory constraints. Given the greater performance impact of layer_o, the final configuration adopted layer_i = 3 and layer_o = 4, balancing computational feasibility with optimal accuracy.

Supplementary Figures

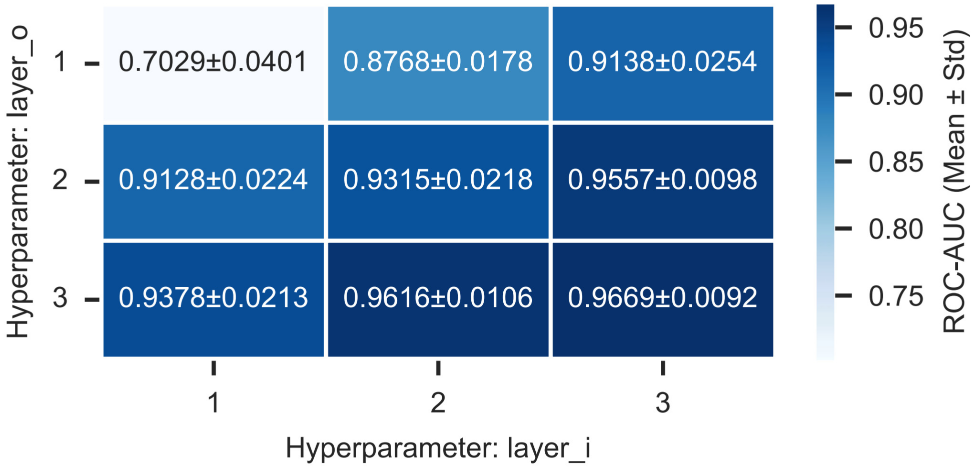

Figure S1 Hypermeter search for layer_i and layer_o.

Supplementary Tables

Table S1 Detailed metrics of hypermeter search for dim1.

Mean ± standard deviation (std) are shown.

dim1	ROC_AUC	PR_AUC	Accuracy	Precision	Recall
2	83.07±11.64(%)	84.97±8.37(%)	76.03±9.01(%)	74.73±9.83(%)	80.56±6.30(%)
4	93.18±1.65(%)	92.41±2.58(%)	87.74±2.84(%)	87.05±4.03(%)	88.82±1.29(%)
8	93.15±5.70(%)	93.68±4.54(%)	87.66±8.67(%)	86.06±11.17(%)	91.88±1.76(%)
16	97.01±0.76(%)	97.08±0.81(%)	93.53±1.08(%)	93.76±1.57(%)	93.29±1.16(%)

Table S2 Detailed metrics of hypermeter search for dim2.

Mean ± standard deviation (std) are shown.

dim2	ROC_AUC	PR_AUC	Accuracy	Precision	Recall
2	95.97±1.20(%)	96.22±1.14(%)	92.40±2.32(%)	91.96±4.62(%)	93.19±0.95(%)
4	96.73±0.97(%)	96.75±1.09(%)	92.08±1.93(%)	91.69±3.77(%)	92.72±1.22(%)
8	96.48±0.52(%)	96.71±0.46(%)	93.18±1.48(%)	92.64±2.24(%)	93.86±1.54(%)
16	96.76±1.10(%)	96.78±1.12(%)	93.36±1.88(%)	94.28±1.67(%)	92.34±3.36(%)

Table S3 Detailed metrics of hypermeter search for layer_i.

Mean ± standard deviation (std) are shown.

layer_i	ROC_AUC	PR_AUC	Accuracy	Precision	Recall
1	93.78±2.13(%)	93.98±2.19(%)	87.35±3.09(%)	86.36±4.40(%)	88.94±1.13(%)
2	96.16±1.06(%)	96.14±1.18(%)	91.84±1.39(%)	92.87±2.27(%)	90.70±2.43(%)
3	96.69±0.92(%)	96.79±0.88(%)	93.13±1.71(%)	93.17±1.49(%)	93.09±2.22(%)
4	96.87±0.79(%)	97.06±0.64(%)	94.09±1.43(%)	94.72±0.75(%)	93.38±2.80(%)

Table S4 Detailed metrics of hypermeter search for layer_o.

Mean ± standard deviation (std) are shown.

layer_o	ROC_AUC	PR_AUC	Accuracy	Precision	Recall
1	91.38±2.54(%)	91.67±2.78(%)	83.97±3.88(%)	84.02±5.27(%)	84.17±3.65(%)
2	95.57±0.98(%)	95.44±1.28(%)	89.86±1.92(%)	93.18±1.75(%)	86.03±3.29(%)
3	96.69±0.92(%)	96.79±0.88(%)	93.13±1.71(%)	93.17±1.49(%)	93.09±2.22(%)
4	97.40±0.73(%)	97.52±0.83(%)	94.04±1.02(%)	94.62±1.93(%)	93.43±1.88(%)

Table S5 Performance comparison of NovelHTI with baseline models on dataset 02.

Mean ± standard deviation (std) are shown.

model	ROC_AUC	PR_AUC	Accuracy	Precision	Recall
NovelHTI	85.36±1.64(%)	87.18±1.19(%)	79.46±1.38(%)	84.25±1.39(%)	72.55±3.94(%)
HAN	78.42±2.10(%)	75.30±1.88(%)	71.20±1.92(%)	74.20±2.05(%)	67.79±2.45(%)
XGBoost	68.95±1.20(%)	65.40±1.55(%)	62.15±1.40(%)	63.50±1.50(%)	60.38±1.65(%)
HTINet2+ERMLPE	51.47±2.96(%)	63.13±2.68(%)	50.39±1.66(%)	50.35±1.60(%)	51.97±3.78(%)
HTINet2+PairRE	50.80±3.72(%)	62.67±2.74(%)	50.35±2.36(%)	50.27±2.34(%)	50.53±4.43(%)
HTINet2+TuckER	50.73±3.45(%)	62.34±2.98(%)	50.51±2.74(%)	50.46±2.68(%)	51.10±4.06(%)
HTINet2+MuRE	50.37±4.05(%)	61.95±2.94(%)	50.59±2.22(%)	50.50±2.32(%)	49.85±5.33(%)
HTINet2+TransR	49.95±3.77(%)	61.83±2.98(%)	49.61±2.34(%)	49.51±2.41(%)	50.01±5.23(%)
HTINet2+ComplEx	49.88±5.27(%)	61.58±4.47(%)	49.81±2.91(%)	49.68±2.74(%)	50.04±6.56(%)
HTINet2+ProjE	49.49±5.20(%)	61.62±3.68(%)	49.75±3.21(%)	49.55±3.29(%)	49.43±7.05(%)
HTINet2+CrossE	49.30±5.10(%)	61.65±4.08(%)	49.49±3.16(%)	49.33±3.27(%)	49.00±6.03(%)

Table S6 Performance comparison of NovelHTI with baseline models on dataset 04.

Mean ± standard deviation (std) are shown.

model	ROC_AUC	PR_AUC	Accuracy	Precision	Recall
NovelHTI	94.86±1.01(%)	95.56±0.64(%)	91.53±0.93(%)	93.35±0.93(%)	89.46±2.17(%)
HAN	86.55±1.42(%)	84.10±1.10(%)	81.25±1.50(%)	82.50±1.60(%)	79.46±1.75(%)
XGBoost	74.20±0.95(%)	72.80±1.12(%)	68.50±1.10(%)	69.80±1.25(%)	66.67±1.35(%)
HTINet2+ComplEx	53.31±5.74(%)	68.52±3.85(%)	51.13±1.96(%)	50.96±2.15(%)	54.31±6.98(%)
HTINet2+TuckER	51.79±6.28(%)	67.35±4.33(%)	50.26±2.52(%)	50.09±2.54(%)	52.12±7.37(%)
HTINet2+ERMLPE	50.18±3.09(%)	66.56±2.25(%)	49.86±1.25(%)	49.85±1.22(%)	49.76±3.75(%)
HTINet2+MuRE	49.61±5.16(%)	65.85±3.85(%)	49.63±2.15(%)	49.51±2.28(%)	49.85±5.91(%)
HTINet2+TransR	49.53±6.36(%)	65.57±4.90(%)	50.27±2.29(%)	50.09±2.29(%)	49.64±7.98(%)
HTINet2+ProjE	49.40±5.36(%)	65.61±3.82(%)	49.29±1.63(%)	49.17±1.62(%)	49.46±6.98(%)
HTINet2+CrossE	48.80±5.69(%)	64.85±3.94(%)	50.49±1.46(%)	50.39±1.43(%)	48.53±7.27(%)
HTINet2+PairRE	46.39±6.08(%)	63.65±4.71(%)	48.60±2.04(%)	48.34±2.22(%)	46.15±7.58(%)

Table S7 Performance comparison of NovelHTI with baseline models on dataset 06.

Mean ± standard deviation (std) are shown.

model	ROC_AUC	PR_AUC	Accuracy	Precision	Recall
NovelHTI	95.04±1.71(%)	95.69±1.28(%)	91.51±2.03(%)	93.17±1.85(%)	89.60±3.36(%)
HAN	87.10±1.65(%)	84.50±1.35(%)	81.80±1.85(%)	83.10±1.95(%)	80.06±2.15(%)
XGBoost	75.15±1.05(%)	73.20±1.25(%)	69.10±1.30(%)	70.40±1.45(%)	67.37±1.55(%)
HTINet2+ComplEx	51.72±5.37(%)	67.45±4.03(%)	50.30±1.97(%)	50.18±1.93(%)	52.40±6.51(%)
HTINet2+MuRE	51.10±4.64(%)	66.90±3.59(%)	50.10±2.11(%)	50.06±2.00(%)	51.83±5.30(%)
HTINet2+TuckER	50.71±6.00(%)	66.47±4.48(%)	50.50±2.28(%)	50.36±2.21(%)	50.16±7.31(%)
HTINet2+CrossE	50.54±4.43(%)	66.50±3.17(%)	49.95±1.62(%)	49.86±1.59(%)	50.15±5.96(%)

HTINet2+ERMLPE	50.02±4.54(%)	66.17±3.43(%)	49.72±1.73(%)	49.62±1.74(%)	49.71±6.35(%)
HTINet2+PairRE	48.33±5.53(%)	65.03±4.17(%)	49.48±2.25(%)	49.31±2.37(%)	48.16±7.35(%)
HTINet2+ProjE	48.19±5.01(%)	64.83±3.66(%)	49.34±2.16(%)	49.20±2.23(%)	47.28±6.88(%)
HTINet2+TransR	48.16±4.79(%)	64.33±4.43(%)	50.25±1.93(%)	50.14±2.00(%)	47.63±6.49(%)
